# Muscle-specific ER-associated degradation maintains postnatal muscle hypertrophy and systemic energy metabolism

**DOI:** 10.1172/jci.insight.170387

**Published:** 2023-09-08

**Authors:** Benedict Abdon, Yusheng Liang, Débora da Luz Scheffer, Mauricio Torres, Neha Shrestha, Rachel B. Reinert, You Lu, Brent Pederson, Amara Bugarin-Lapuz, Sander Kersten, Ling Qi

**Affiliations:** 1 Department of Molecular & Integrative Physiology, University of Michigan Medical School, Ann Arbor, Michigan, USA.; 2 Department of Anatomy, Institute of Biomedical Sciences, University of Sao Paulo, Sao Paulo, Brazil.; 3 Division of Metabolism, Endocrinology & Diabetes, Department of Internal Medicine, University of Michigan Medical School, Ann Arbor, Michigan, USA.; 4 Nutrition Metabolism and Genomics group, Wageningen University, Wageningen, Netherlands.; 5 Department of Biological Chemistry, University of Michigan Medical School, Ann Arbor, Michigan, USA.

**Keywords:** Cell Biology, Muscle Biology, Cell stress, Protein misfolding, Skeletal muscle

## Abstract

The growth of skeletal muscle relies on a delicate equilibrium between protein synthesis and degradation; however, how proteostasis is managed in the endoplasmic reticulum (ER) is largely unknown. Here, we report that the SEL1L-HRD1 ER-associated degradation (ERAD) complex, the primary molecular machinery that degrades misfolded proteins in the ER, is vital to maintain postnatal muscle growth and systemic energy balance. Myocyte-specific SEL1L deletion blunts the hypertrophic phase of muscle growth, resulting in a net zero gain of muscle mass during this developmental period and a 30% reduction in overall body growth. In addition, myocyte-specific SEL1L deletion triggered a systemic reprogramming of metabolism characterized by improved glucose sensitivity, enhanced beigeing of adipocytes, and resistance to diet-induced obesity. These effects were partially mediated by the upregulation of the myokine FGF21. These findings highlight the pivotal role of SEL1L-HRD1 ERAD activity in skeletal myocytes for postnatal muscle growth, and its physiological integration in maintaining whole-body energy balance.

## Introduction

Skeletal muscle is not only indispensable for physical movement, but also plays a major role in regulating whole-body energy and protein metabolism (1–3). Consequently, preserving muscle mass and function is essential to maintain systemic energy balance. Muscle mass is determined by a delicate balance of protein synthesis and degradation (proteostasis), where the rate of synthesis exceeds the rate of degradation to promote cellular growth. The most distinct physiological example of this is during postnatal development, where muscle size significantly increases to achieve the appropriate mass necessary for adult function i.e., contraction, force generation, and energy production. Multiple signaling pathways that regulate proteostasis, including mTORC1, IGF1/PI3K/AKT, myostatin/Smad3, autophagy, and the ubiquitin-proteosome pathway have been well established to contribute to muscle mass during this period (4–8). However, the importance of endoplasmic reticulum–associated degradation (ERAD), the principal mechanism in the ER responsible for directly managing ER protein turnover, in muscle biology remains unexplored.

ERAD is responsible for recruiting and retrotranslocating misfolded ER proteins for cytosolic proteasomal degradation (9–12). Among dozens of ER-resident E3 ligase protein complexes in mammals, the SEL1L-HRD1 protein complex represents the most conserved branch of ERAD, where the single-span transmembrane protein SEL1L acts as a critical cofactor for the stability and function of the multispan transmembrane protein E3 ubiquitin ligase HRD1 (13–15). Global and inducible *Sel1L*- (16, 17) or *Hrd1*-knockout (18, 19) mice exhibit embryonic and premature lethality, respectively, demonstrating the indispensable role of SEL1L and HRD1 during development. Subsequent studies using cell type–specific mouse models have revealed the roles of SEL1L and HRD1 in regulating energy metabolism, immune/stem cell function, food intake, and water balance (11, 17, 20–34). However, the physiological significance of SEL1L-HRD1 ERAD in skeletal muscle is so far unknown.

In this study, we show that skeletal myocyte–specific SEL1L-HRD1 ERAD is essential for maintaining muscle mass and whole-body energy metabolism in young developing mice (4–12 weeks of age). While the loss of SEL1L does not immediately impact muscle growth after birth, it significantly impairs myofiber growth and alters systemic metabolism during the rapid growth phase of postnatal development. Mice with myocyte-specific SEL1L deficiency exhibit enhanced insulin sensitivity, increased adipose beigeing, and resistance to diet-induced obesity. This metabolic adaptation is partially caused by the induction of FGF21 in the myocytes and resulting systemic hyper-FGF21-nemia.

## Results

### Expression of SEL1L-HRD1 ERAD in skeletal muscles.

**Figure 1:**
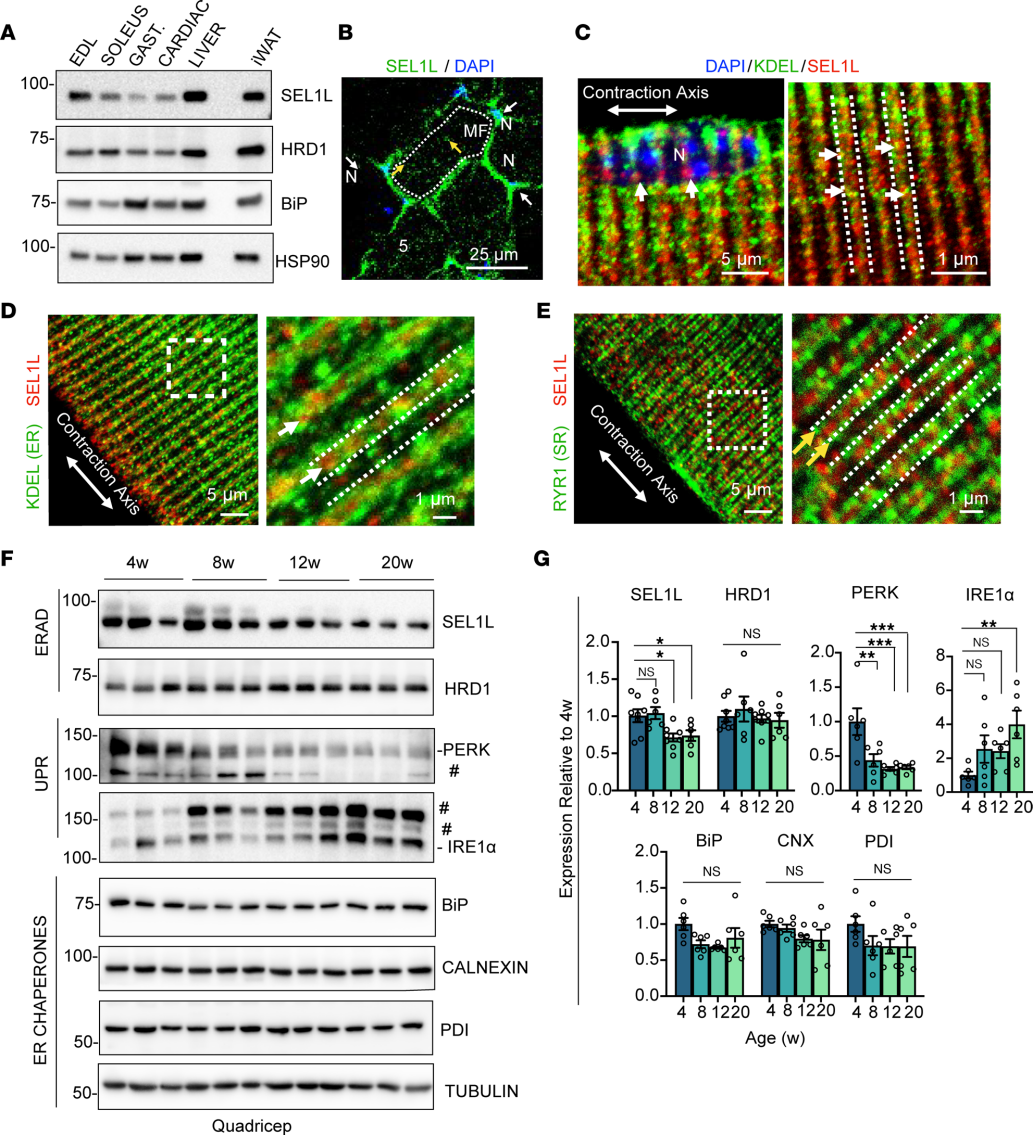
SEL1L-HRD1 ERAD expression in muscles. (**A**) Representative Western blot of SEL1L, HRD1, and BiP in indicated mouse tissues (*n* = 3 mice). (**B**) Representative confocal image of tibialis anterior cross sections stained for SEL1L and DAPI (*n* = 3 mice). Dotted line indicates individual myofiber (MF). N, nucleus. White arrows indicate perinuclear SEL1L expression. Yellow arrows indicate interfibrillar SEL1L expression. (**C**–**E**) Representative confocal images of isolated EDL myofibers stained for SEL1L, ER marker KDEL, or the SR marker ryanodine receptor 1 (RYR1) (*n* = 3 mice). White arrows indicate regions of KDEL and SEL1L colocalization. Dashed line represents intermyofibrillar ER localization (**C** and **D**). Yellow arrows in **E** indicate RYR1/SR localization, with dashed lines indicating intermyofibrillar SR location. Boxes indicate high-magnification inset. (**F** and **G**) Western blot analysis and quantitation of SEL1L-HRD1 and ER chaperones in quadriceps muscle of 4-, 8-, 12-, and 20-week-old WT C57BL/6J mice (*n* = 6–8 mice per time point). Data presented as mean ± SEM, normalized to the 4-week time point. NS, *P* > 0.05; *P < 0.05; **P < 0.01; ***P < 0.001 determined by 1-way ANOVA with Dunnett’s multiple-comparison test.

We first determined the relative protein expression of SEL1L-HRD1 ERAD in different adult mouse organs. Generally, the levels of SEL1L and HRD1 proteins were lower in striated muscles compared with liver and inguinal white adipose tissue (iWAT) (Figure 1A). Among the muscles examined, the highest expression of SEL1L was observed in the primarily fast-twitch muscle fiber, extensor digitorum longus (EDL), whereas HRD1 protein levels remained relatively consistent across muscle tissues (Figure 1A). To investigate the subcellular localization of SEL1L, we analyzed cross sections of the tibialis anterior muscle and observed strong perinuclear staining of SEL1L surrounding individual myofibers (Figure 1B, white arrows). Additionally, we observed small SEL1L puncta deeper within the myofiber situated between the myofibrils (yellow arrows). Using isolated myofibers, we confirmed that SEL1L was not only localized in the perinuclear region (Figure 1C), but was distributed perpendicular to the length of the fiber in repeated intervals in a highly organized pattern (Figure 1C). Moreover, SEL1L exhibited greater overlap with the canonical ER marker KDEL compared with the sarcoplasmic reticulum (SR) marker ryanodine receptor 1 (RYR1) (arrows; Figure 1, D and E).

We next measured the temporal expression pattern of ERAD and proteins involved in ER homeostasis in muscles during the transition between young (4 weeks of age) and adult mice (20 weeks of age) (Figure 1, E and F), a critical developmental phase characterized by rapid protein synthesis (35). SEL1L protein expression exhibited its highest levels at 4 weeks of age and gradually decreased as the mouse developed into adulthood. In contrast, HRD1 and several ER chaperones (BiP, calnexin, and protein disulfide isomerase [PDI]) remained relatively constant throughout this developmental period. The expression of the unfolded protein response (UPR) sensor PERK was also highest at 4 weeks of age and progressively declined with age, while IRE1α protein displayed the opposite trend, being lowest at 4 weeks of age and gradually increasing with age. Collectively, these findings suggest a highly dynamic nature of the ER in muscle during early developmental stages, where SEL1L-HRD1 ERAD may contribute to the regulation of myocyte function.

### Myocyte-specific SEL1L deficiency alters body growth and systemic energy metabolism.

To elucidate the significance of SEL1L-HRD1 ERAD in skeletal muscle, we developed a skeletal myocyte–specific *Sel1L*-knockout (*Sel1L^MLC^*) mouse model by crossing *Sel1L*-floxed (*Sel1L^fl/fl^*) mice (17) with transgenic mice expressing Cre recombinase under the endogenous myosin light chain locus (MLC^Cre^) (36). The MLC promoter exhibits activity at embryonic day 9.5 to 10.5 and primarily targets fast-twitch fibers in skeletal muscles (36). In *Sel1L^MLC^* mice compared with their *Sel1L^fl/fl^* (WT) littermates, both mRNA and protein expression of SEL1L was significantly reduced in skeletal muscle, while no notable changes were observed in WAT or liver (Supplemental Figure 1A; supplemental material available online with this article; https://doi.org/10.1172/jci.insight.170387DS1). For unknown reasons, HRD1 mRNA and protein remained unaltered (Supplemental Figure 1, A–D), despite previous studies demonstrating that HRD1 protein stability depends on SEL1L in vivo (17, 20, 21, 23, 24, 28, 37, 38).

**Figure 2:**
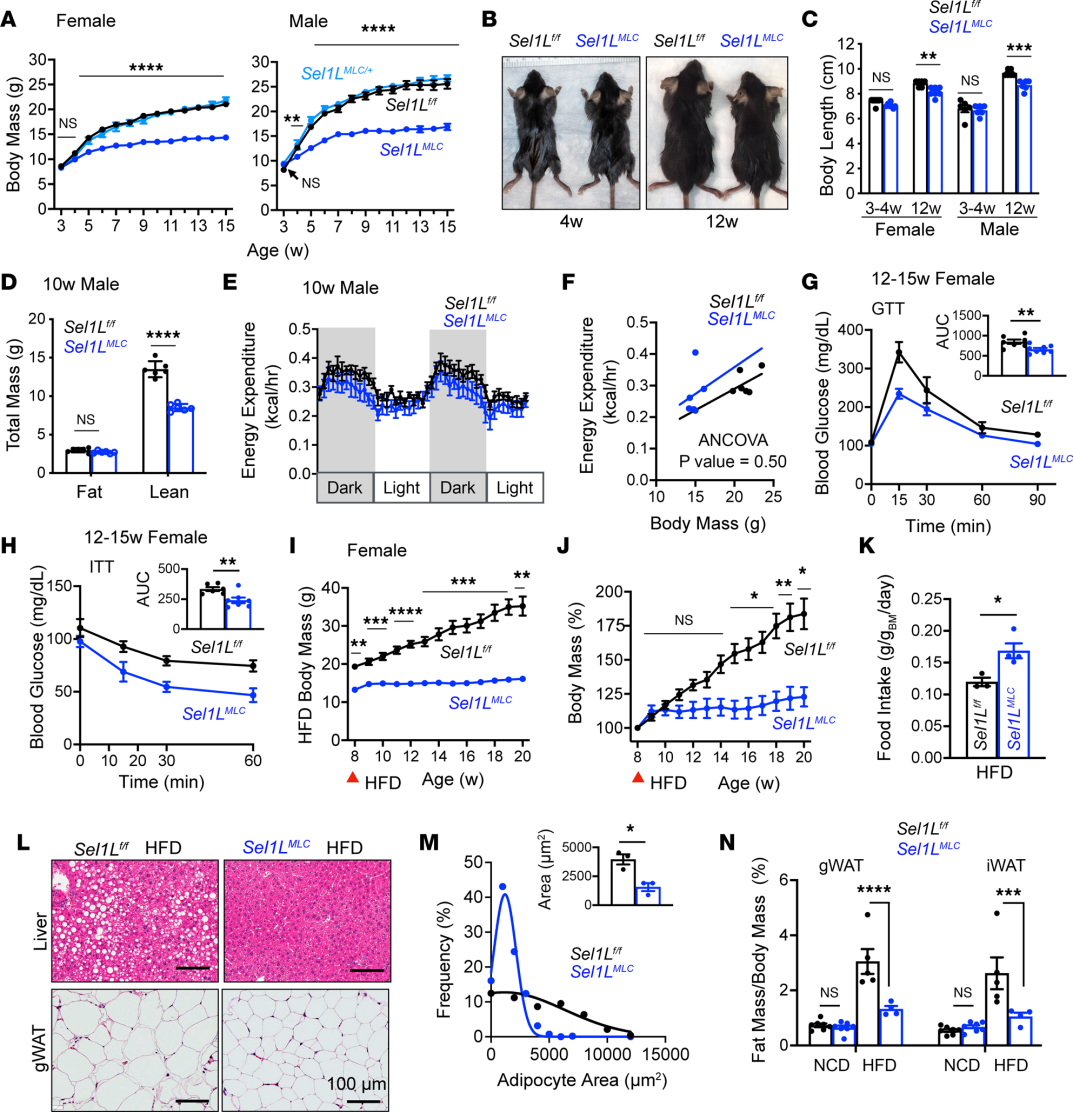
Myocyte-specific SEL1L deficiency alters body growth and systemic energy metabolism. (**A**) Body mass curves of female and male mice (*n* = 6–36 mice per genotype/time point). Statistical comparison is between *Sel1L^fl/fl^* (WT) and *Sel1L^MLC^* mice. (**B**) Representative photos of male mice. (**C**) Body length of male and female mice (*n* = 6–7 mice per genotype/time point). (**D**) Fat and lean mass of male mice (*n* = 6 mice per genotype). (**E**) Raw energy expenditure and (**F**) ANCOVA-adjusted energy expenditure (*n* = 6 mice per genotype). (**G**) Blood glucose curve and area under curve (AUC) for intraperitoneal glucose tolerance test (IPGTT) of 12- to 15-week-old female mice (*n* = 7–8 mice per genotype). (**H**) Blood glucose curve and area under curve (AUC) for IPITT of 12- to 15-week-old female mice (*n* = 6–7 mice per genotype). (**I**) Body mass curve of female mice during high-fat diet (HFD) feeding (*n* = 5–9 mice per genotype/time point). Red arrow indicates start of HFD feeding (day 0). (**J**) Body mass (percentage) relative to day 0 during HFD feeding (*n* = 5–9 mice per genotype/time point). (**K**) HFD food intake (*n* = 3–4 mice). (**L**) Representative H&E staining of peripheral tissues after 12 weeks of HFD (*n* = 5). (**M**) Adipocyte area of mice fed HFD (*n* = 3 mice per genotype, ~150–200 adipocytes measured per animal). (**N**) Fat mass of mice under normal chow diet (NCD) and HFD (*n* = 4–7 mice per treatment/genotype). Data presented as mean ± SEM. NS, *P* > 0.05; *P < 0.05; **P < 0.01; ***P < 0.001; ****P < 0.0001 determined by mixed-effects analysis (repeated-measure ANOVA) with Tukey’s multiple-comparison test (**A**, **I**, and **J**), 2-way ANOVA with Tukey’s multiple-comparison test (**C** and **N**), 2-tailed, unpaired *t* test (**D**,**G**,**H**, **K**, and **M**), or ANCOVA (**F**).

During the initial 3 to 4 weeks of postnatal development under normal chow diet (13% kcal from fat), there were no discernible differences in body mass and size between *Sel1L^MLC^* mice and their WT littermates. However, by 12 weeks of age, *Sel1L^MLC^* mice exhibited a notable reduction in size, measuring approximately 10% shorter and 30% lighter in terms of body mass compared with WT and heterozygous *Sel1L^MLC/+^* littermates (Figure 2, A–C). Analysis of body composition revealed that the decreased overall mass in *Sel1L^MLC^* mice was primarily attributable to a reduction in lean mass (Figure 2D). Histological examination of the liver, gonadal WAT, and pancreas displayed no evident differences between genotypes under basal conditions (Supplemental Figure 1E). Additionally, various physiological parameters, including energy expenditure, internal body temperature, locomotor activity, food intake, and respiratory exchange ratio (RER), showed no significant disparities between WT and *Sel1L*^MLC^ mice during basal resting conditions (Figure 2, E and F, and Supplemental Figure 1, F–J). These observations were consistent for both males and females.

Blood glucose and lactate measurements obtained under ad libitum feeding conditions did not reveal any significant difference between WT and *Sel1L^MLC^* mice (Supplemental Figure 1K). However, glucose (GTT) and insulin tolerance tests (ITT) indicated that *Sel1L^MLC^* mice displayed improved glucose clearance compared with WT mice (Figure 2, G and H), indicating enhanced insulin sensitivity. To further evaluate this, both male and female WT and *Sel1L^MLC^* mice were subjected to a 12-week high-fat diet (HFD) regimen (60% kcal from fat). *Sel1L^MLC^* mice exhibited significant resistance to diet-induced obesity (Figure 2, I and J, and Supplemental Figure 2, A and B), despite consuming a greater amount of food on average than WT mice (Figure 2K). Compared with WT littermates, *Sel1L^MLC^* mice displayed minimal hepatic steatosis, reduced white adipocyte size (Figure 2L), and decreased WAT mass (Figure 2, M and N, and Supplemental Figure 2C). Furthermore, *Sel1L^MLC^* mice exhibited lower ad libitum blood glucose and serum insulin than WT littermates under the HFD conditions, consistent with their enhanced insulin sensitivity (Supplemental Figure 2, D and E). These findings suggest that a systemic metabolic reprogramming occurs in mice lacking SEL1L in skeletal muscle, contributing to their resistance to diet-induced obesity.

### Myocyte-specific SEL1L is required for hypertrophic muscle growth.

**Figure 3:**
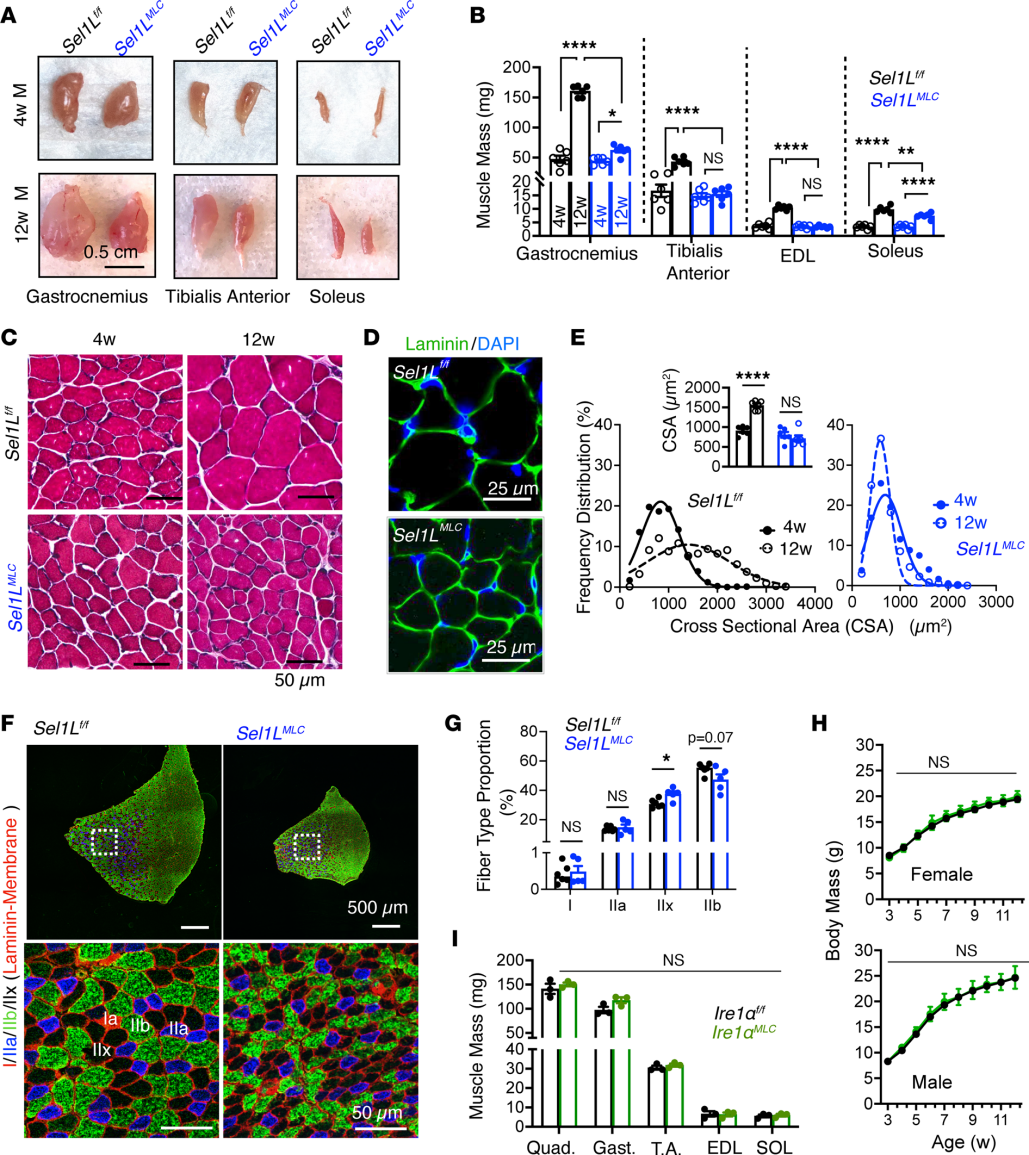
Postnatal hypertrophic growth of skeletal muscle requires SEL1L-HRD1 ERAD. (**A**) Representative photos of hindlimb skeletal muscle tissue in mice at 4 and 12 weeks old. (**B**) Comparison of glycolytic (gastrocnemius, tibialis anterior, EDL) and oxidative muscle (soleus) mass of 4- and 12-week-old male mice (*n* = 6 per genotype/time point). (**C**) Representative H&E staining of tibialis anterior muscle in 4- and 12-week-old mice (*n* = 6 mice per genotype). (**D**) Representative confocal image of laminin staining of 8-week-old tibialis anterior muscle (*n* = 4 mice per genotype). (**E**) Frequency distribution of muscle cross-sectional area of 4- and 12-week-old tibialis anterior muscle with quantitation (*n* = 6 mice per genotype/time point, ~100–200 fibers per mouse). (**F**) Fiber type analysis of myosin heavy chain (MyHC) subtypes, type I (red), type IIa (blue), type IIx (unstained/black), and type IIb (green), with laminin staining the myofiber membrane (*n* = 5–6 mice per genotype). (**G**) Quantitation of fiber type percentage of tibialis anterior muscle from 8-week-old mice (*n* = 5–6 mice per genotype). (**H**) Body mass curves of male and female *Ire1α^fl/fl^* and *Ire1α^MLC^* mice (*n* = 4–28 per genotype/sex/time point). (**I**) Comparison of glycolytic (quadriceps, gastrocnemius, tibialis anterior [T.A.], EDL) and oxidative muscle (soleus [SOL]) mass of 15-week-old male mice (*n* = 3 mice per genotype). Data presented as mean ± SEM. NS, *P* > 0.05; *P < 0.05; **P < 0.01; ****P < 0.0001 determined by 2-way ANOVA with Tukey’s multiple-comparison test (**B** and **E**), 2-tailed, unpaired *t* test (**G** and **I**), or mixed-effects analysis (repeated-measure ANOVA) (**H**).

To investigate the underlying cause of reduced body growth and lean mass, we examined muscle growth from 4 to 12 weeks of age. In WT mice, the mass of both fast-twitch muscles (gastrocnemius, tibialis anterior, EDL) and the slow-twitch soleus muscle significantly increased during this period (Figure 3, A and B). In contrast, muscles from *Sel1L^MLC^* mice reached a plateau and remained relatively unchanged in size between the juvenile and adult stages (Figure 3, A and B). Importantly, the muscles of *Sel1L^MLC^* mice did not experience a loss of mass but rather exhibited a failure to grow during this developmental period (Figure 3B). Additionally, the quadriceps muscle in *Sel1L^MLC^* mice did not scale proportionally with increases in body mass (and to some extent, age) (*r*^2^ = 0.4495), in contrast to the scaling observed in WT mice (*r*^2^ = 0.8938) (Supplemental Figure 3, A and B). However, this effect was less pronounced in the soleus muscle (Supplemental Figure 3, A and B), aligning with the notion that MLC^Cre^ selectively deletes SEL1L in muscles containing predominately fast-twitch fibers rather than slow-twitch fibers. In contrast, the liver exhibited proportional growth with body mass (Supplemental Figure 3, A and B). Collectively, these findings indicate that *Sel1L^MLC^* mice demonstrate an impaired muscle growth rate.

Immunofluorescence and histological analyses of muscle tissue revealed that the reduced muscle mass in *Sel1L^MLC^* mice was primarily due to diminished myofiber size (Figure 3, C and D). In contrast to WT mice, *Sel1L^MLC^* mice did not exhibit hypertrophic growth of the tibialis anterior muscle between 4 and 12 weeks of age (Figure 3E). Fiber-typing analysis revealed no significant difference in the proportions of oxidative type I, intermediate type IIa, or glycolytic type IIb fibers between the 2 groups. However, *Sel1L^MLC^* mice displayed a slight increase in glycolytic type IIx fibers (Figure 3, F and G), indicating a subtle influence of SEL1L loss on muscle fiber types.

Unlike *Sel1L^MLC^* mice, mice with myocyte-specific deletion of IRE1α, a key UPR sensor (39), appeared largely unaffected in terms of postnatal growth and muscle development during the same growth period (Figure 3, H and I, and Supplemental Figure 3, C and D). These findings collectively demonstrate that SEL1L-HRD1 ERAD plays a role in regulating postnatal hypertrophic muscle growth.

### Loss of myocyte-specific SEL1L alters global proteostasis.

**Figure 4:**
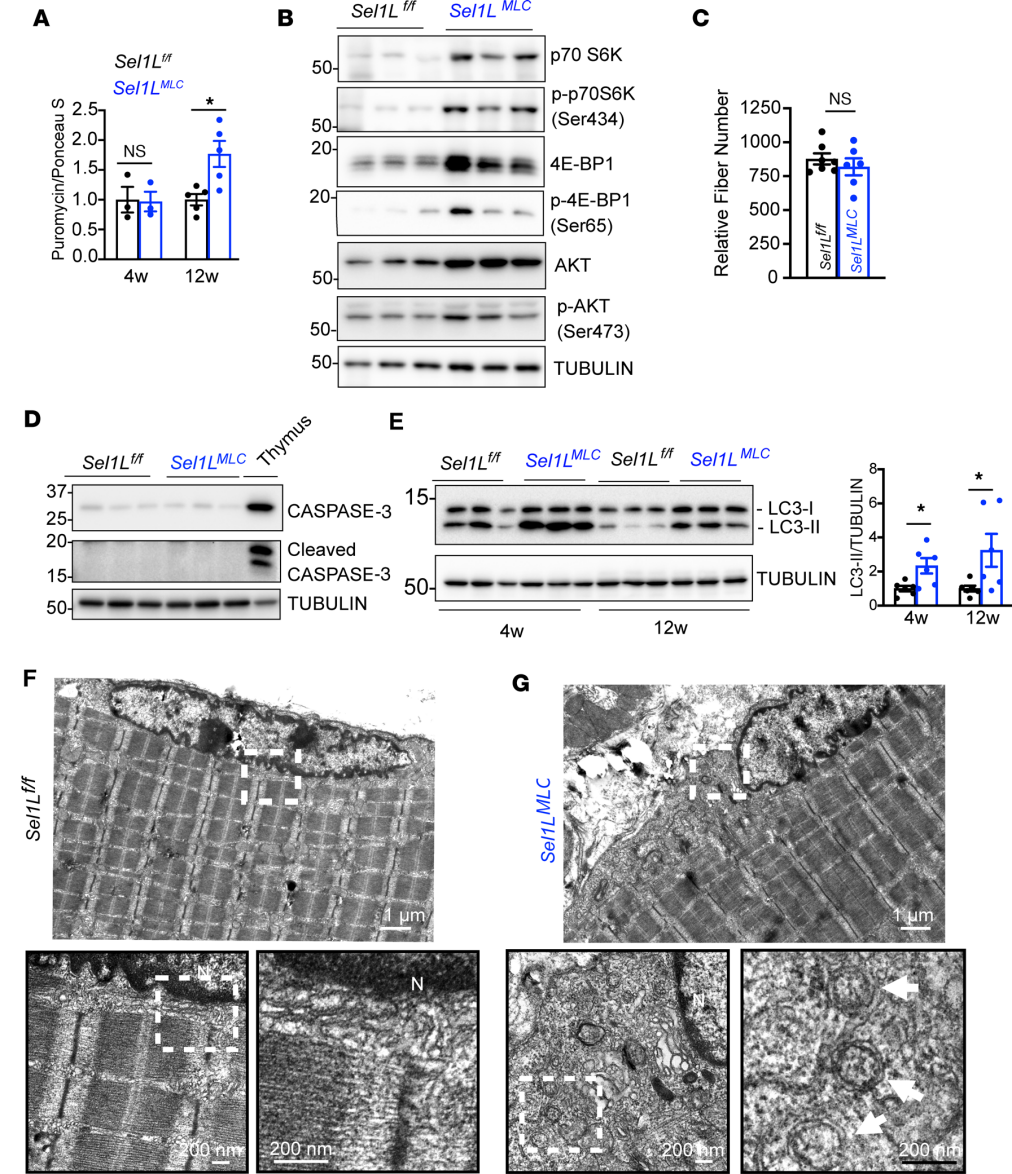
Loss of myocyte-specific SEL1L alters global proteostasis in muscle. (**A**) Quantitation of puromycin incorporation in 4- and 12-week-old quadriceps muscle (*n* = 3–5 mice per genotype/age). (**B**) Representative Western blot of total/phosphorylated forms of AKT, 4EBP1, and p70S6K in 12-week-old quadriceps muscle (*n* = 6 mice per genotype). (**C**) Relative fiber number in adult (12–20 weeks old) tibialis anterior muscle (*n* = 6–7 mice per genotype). (**D**) Western blot of cleaved and uncleaved caspase-3. Protein lysates from the thymus were used as a positive control (*n* = 3 mice per genotype). (**E**) Western blot and quantitation of LC3 lipidation in 4- and 12-week-old muscle (*n* = 6 mice per genotype/time point). (**F** and **G**) Representative TEM image of 8-week-old tibialis anterior muscle from *Sel1L^fl/fl^* (**F**) and *Sel1L^MLC^* (**G**) mice (*n* = 2 per genotype). White box indicates higher magnification insets. White arrows indicate autophagosomes. No abnormal autophagosomes were observed in WT (*Sel1L^fl/fl^*) muscle. N, nucleus. Data presented as mean ± SEM. NS, *P* > 0.05; *P < 0.05 determined by 2-tailed, unpaired *t* test (**A**, **C**, and **E**).

To investigate the molecular basis of the impaired muscle growth, we examined proteostatic pathways known to influence muscle development and growth. Expression analysis of genes involved in myogenesis (*MyoD*, *MyoG*, and *Pax7*) showed no significant differences between *Sel1L^MLC^* and WT muscle, indicating that muscle development and differentiation were not affected in *Sel1L^MLC^* muscle (Supplemental Figure 4A). To assess global protein synthesis, we employed in vivo surface sensing of translation (SUnSET) analysis (37) and found that it was comparable between *Sel1L^MLC^* and WT mice at 4 weeks of age, but increased in *Sel1L^MLC^* mice at 12 weeks of age (Figure 4A and Supplemental Figure 4B). Total protein levels as well as activating phosphorylation of the Akt/4EBP1/S6K axis, known regulators of protein synthesis through mTOR signaling (5, 40), were all increased, suggesting that global protein synthesis was not impaired in *Sel1L^MLC^* muscle (Figure 4B and Supplemental Figure 4C). Thus, we conclude that the lack of growth in *Sel1L^MLC^* muscle is not attributable to a general reduction in protein synthesis.

Moreover, no significant differences in fiber number were observed between WT and *Sel1L^MLC^* mice (Figure 4C), excluding muscle turnover as a contributing factor in the growth phenotype. There was also no increase in caspase-3 cleavage, indicating the absence of apoptosis in *Sel1L^MLC^* muscle (Figure 4D). However, Western blot analysis revealed elevated levels of microtubule-associated protein light chain 3 II (LC3-II) in *Sel1L^MLC^* muscle at both 4 and 12 weeks of age (Figure 4E), indicating enhanced autophagic activity. Notably, the elevated LC3-II levels in *Sel1L^MLC^* muscle remained unresponsive to colchicine treatment (38) (Supplemental Figure 4D), suggesting that basal autophagy was already maximally elevated. Additionally, autophagosome formations, characterized by double membrane structures, were observed in the perinuclear regions of *Sel1L^MLC^* tibialis anterior muscle but not in WT muscle (Figure 4, F and G). These data suggest that robust proteostatic changes occur in response to SEL1L deficiency in muscle, including compensatory increases in protein synthesis and enhancement of protein degradation through autophagy. Thus, these data support the integration of SEL1L-HRD1 ERAD with global proteostasis in skeletal muscle to maintain skeletal muscle growth.

### SEL1L is required to maintain muscle ER homeostasis under basal conditions.

**Figure 5:**
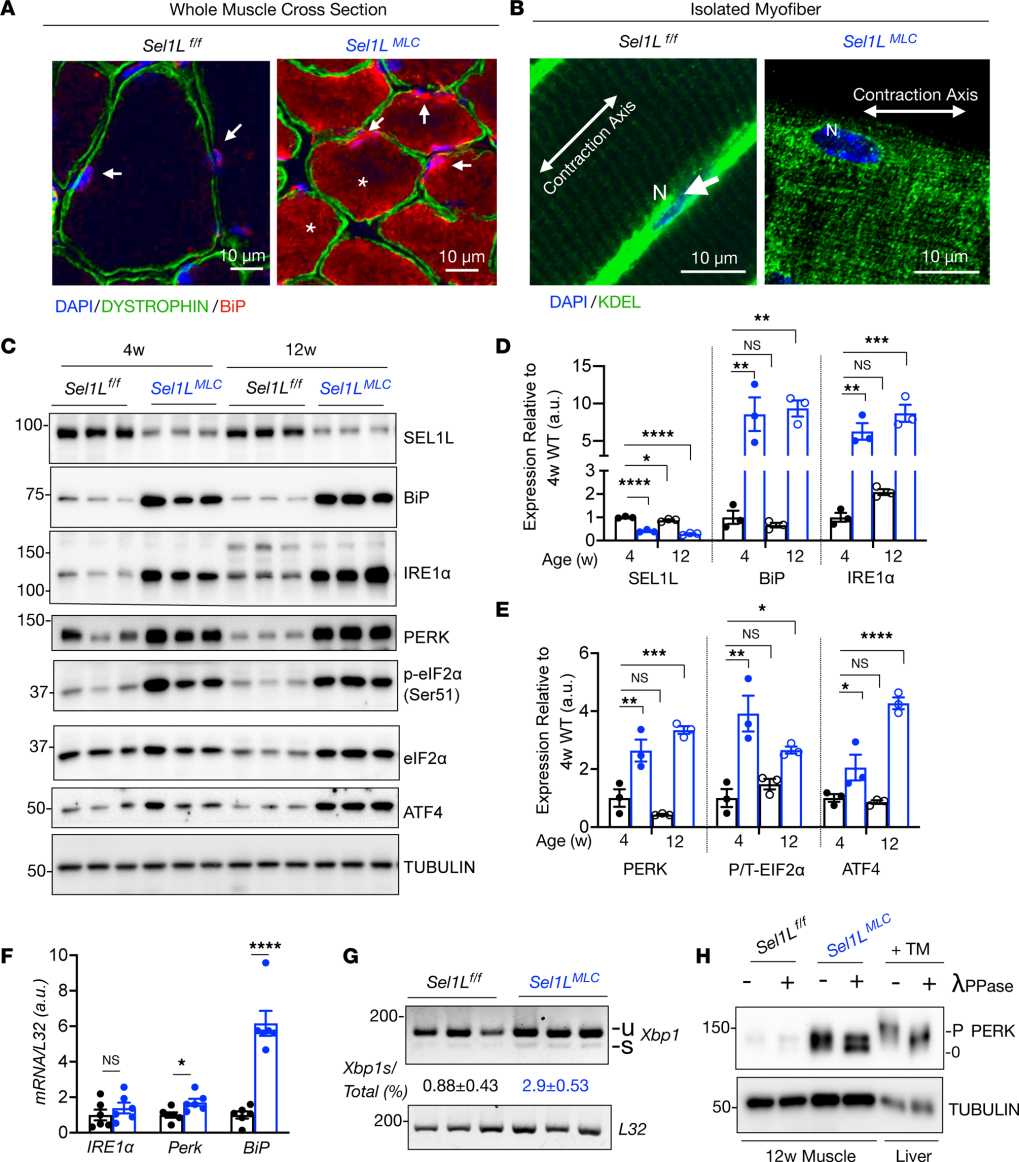
SEL1L is required to maintain muscle ER homeostasis. (**A**) Representative confocal image of tibialis anterior muscle cross section stained for sarcolemma marker dystrophin and ER chaperone BiP. White arrows, perinuclear ER; asterisks, intermyofibrillar ER (*n* = 3 mice per genotype). (**B**) Representative confocal image of isolated EDL myofiber stained with ER marker KDEL (*n* = 5 mice per genotype). White arrows indicate perinuclear ER. (**C**) Western blot analysis and quantitation (**D** and **E**) of ER homeostasis proteins in 4- and 12-week-old muscle (*n* = 3 mice per genotype/time point). (**D**) Quantitation of **C** in 4- and 12-week-old muscle. (**E**) Quantitation of integrated stress response regulators from **C**: PERK, p-eIF2α relative to total eIF2α, and ATF4. (**F**) RT-qPCR of *Ire1α*, *Perk*, and *BiP* in WT and *Sel1L^MLC^* quadriceps muscle (*n* = 5–6 mice per genotype). (**G**) RT-qPCR analysis of *Xbp1* in quadriceps muscle normalized to *L32*. “u” and “s” represent unspliced and spliced forms, respectively (*n* = 6 mice per genotype). (**H**) Western blot of PERK in λ-phosphatase–treated (λPPase-treated) muscle lysates of *Sel1L^fl/fl^* and *Sel1L^MLC^* mice. Livers from tunicamycin-treated (1 mg/kg i.p.) mice served as positive control (*n* = 2 mice per treatment). Data presented as mean ± SEM. NS, *P* > 0.05; *P < 0.05; **P < 0.01; ***P < 0.001; ****P < 0.0001 determined by 2-way ANOVA with Dunnett’s multiple-comparison test (**D** and **E**), or 2-tailed, unpaired *t* test (**F** and **G**).

We next determined how loss of SEL1L affects ER homeostasis in skeletal muscle. Confocal imaging of tibialis anterior cross sections revealed that the ER chaperone, BiP, was increased in a majority of myofibers in *Sel1L^MLC^* muscle (Figure 5A) and expanded into the interfibrillar spaces in *Sel1L^MLC^* muscle (asterisks). In contrast to *Sel1L^MLC^* muscle, BiP in WT muscle mainly localized to the perinuclear regions (arrows, Figure 5A). In support of these findings, we observed similar changes in ER architecture using isolated myofibers (Figure 5B), and a similar increase in BiP protein levels using Western blot analysis (Figure 5, C and D). In line with previous reports identifying IRE1α as an endogenous substrate of ERAD (27), loss of SEL1L significantly increased IRE1α protein levels, but did not affect *IRE1α* mRNA (Figure 5, C, D, and F). RT-PCR analysis of *Xbp1* mRNA splicing, an event catalyzed by activated IRE1α, revealed a 3-fold increase in *Sel1L^MLC^* mice (Figure 5G). Similarly, PERK, another UPR sensor and integrated stress response (ISR) activator, accumulated and became hyperphosphorylated in *Sel1L^MLC^* muscle (Figure 5, C, E, and H). Consistently, phosphorylation, and activation of its downstream effector eIF2α, and protein levels of its transcription factor ATF4 were increased in *Sel1L^MLC^* muscle (Figure 5, C and E). Importantly, the protein levels of UPR ER markers were comparable between 4- and 12-week-old *Sel1L^MLC^* muscles (Figure 5, C–E), indicating that ER proteostasis in *Sel1L^MLC^* muscle does not worsen with age.

**Figure 6:**
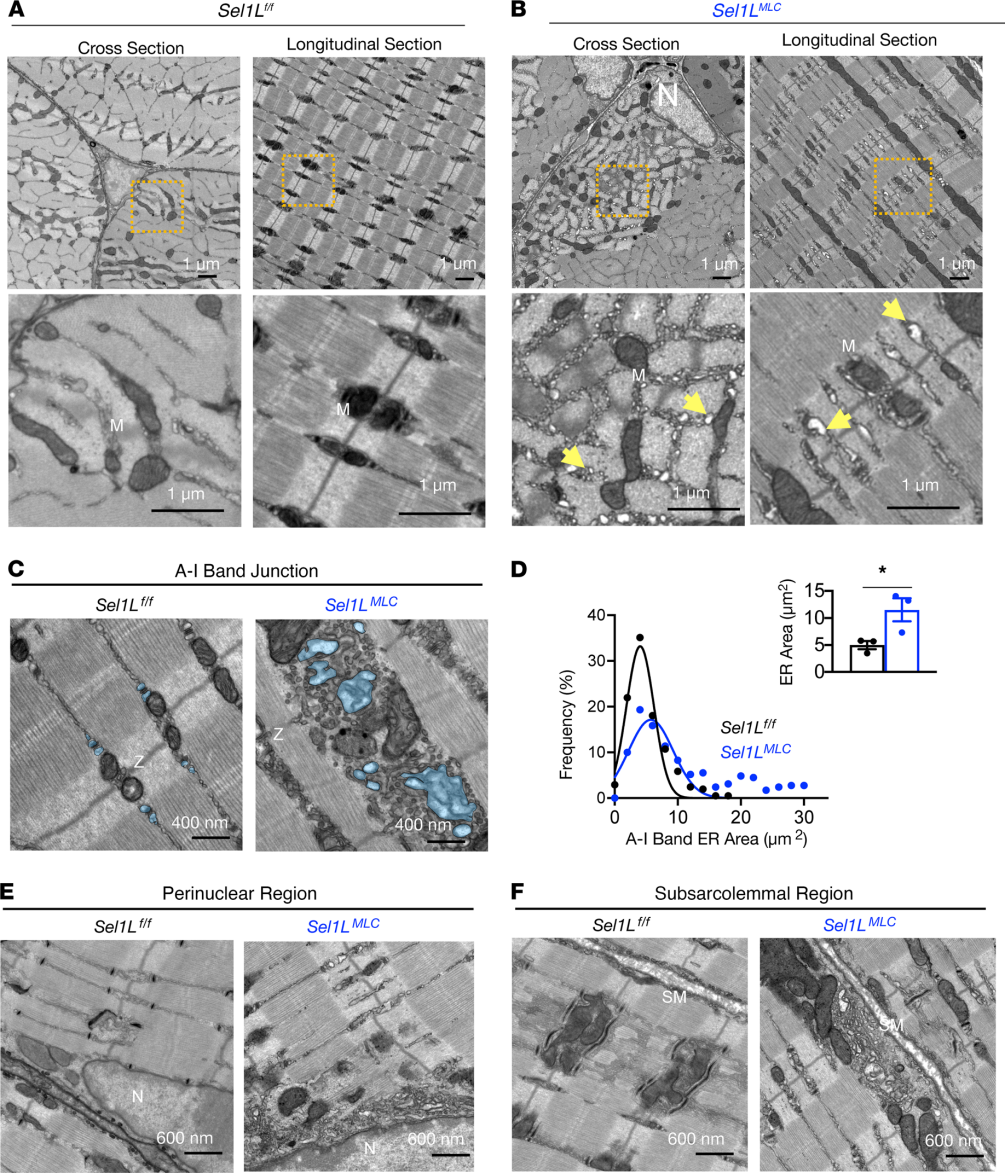
SEL1L deletion results in extensive ER remodeling in muscle. (**A** and **B**) Representative TEM images of WT (**A**) and *Sel1L^MLC^* (**B**) EDL muscle in longitudinal and cross-sectional planes (*n* = 3 mice per genotype). Orange boxes indicate low-magnification inset. Yellow arrows indicate regions of expanded ER/SR membranes. N, nucleus; M, mitochondria. (**C**) Representative image of EDL at the A-I band junction. Blue shades mark the ER. (**D**) Frequency (%) of individual intermyofibrillar SR/ER membrane area (*n* = 3 per genotype). Blue overlay in **C** is representative of regions of interest chosen for quantitation. (**E** and **F**) Representative TEM images of perinuclear (**E**) and subsarcolemmal regions (**F**) in EDL muscle. SM, sarcolemma membrane; N, nucleus. Data presented as mean ± SEM. *P < 0.05 determined by 2-tailed, unpaired *t* test (**D**).

Ultrastructure analysis of EDL muscle revealed distinct differences between *Sel1L^MLC^* and WT. While WT EDL muscle displayed highly organized myofibrils with densely packed organelles (Figure 6A), *Sel1L^MLC^* muscle exhibited large expansions of ER/SR-like structures within the myofibrils (arrows, Figure 6B). Quantitative analysis showed a significant increase in ER area along the Z disc in the A-I band junction (Figure 6, C and D). Furthermore, ER expansions were observed in the perinuclear and subsarcolemmal regions (Figure 6, E and F), indicating widespread ER expansion throughout the muscle fiber. These findings provide evidence that SEL1L-HRD1 ERAD plays a crucial role in maintaining ER proteostasis in muscle during this developmental phase.

### SEL1L deficiency induces FGF21 secretion from skeletal muscle.

**Figure 7:**
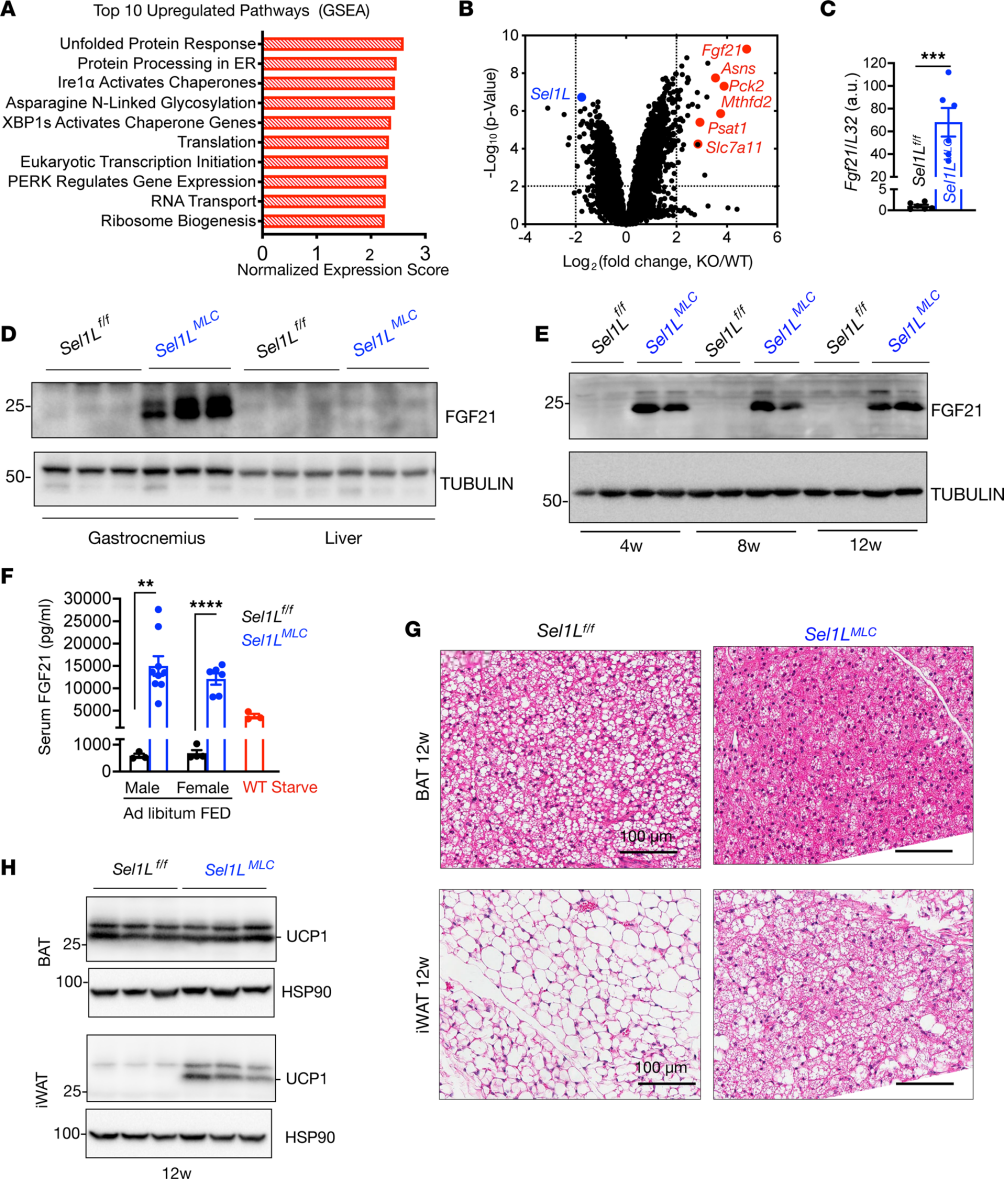
SEL1L deficiency induces FGF21 secretion from skeletal muscle. (**A**) Gene set enrichment analysis (GSEA) of upregulated pathways in female 8-week-old gastrocnemius muscle (*n* = 3 mice per genotype). (**B**) Volcano plot of transcriptomics data from 8-week-old gastrocnemius muscle (*n* = 3 mice per genotype). (**C**) qPCR analysis of *Ffg21* in the gastrocnemius muscle of 4- to 8-week-old female mice (*n* = 6 mice per genotype). (**D**) Western blot of FGF21 in the gastrocnemius muscle and liver from mice under ad libitum conditions (*n* = 3 mice per genotype). (**E**) Western blot of FGF21 in the gastrocnemius muscle from mice under ad libitum conditions at 4, 8, and 12 weeks (*n* = 2 mice per genotype/time point). (**F**) Blood serum levels of FGF21 in adult male and female mice under ad libitum feeding conditions and serum FGF21 levels in WT animals starved for 24 hours (*n* = 3–8 mice per genotype). (**G**) Representative H&E staining of brown adipose tissue (BAT, top) and inguinal white adipose tissue (iWAT, bottom) from male 12-week-old *Sel1L^fl/fl^* and *Sel1L^MLC^* mice (*n* = 4 per genotype). (**H**) Western blot of UCP1 in BAT and iWAT from 12-week-old male mice (*n* = 3 mice per genotype). Data presented as mean ± SEM. **P < 0.01; ***P < 0.001; ****P < 0.0001 determined by 2-tailed, unpaired *t* test (**C** and **F**).

To investigate the systemic effects of myocyte SEL1L-HRD1 ERAD function, we conducted unbiased transcriptomics analysis of the gastrocnemius muscle in 8-week-old mice. Gene set enrichment analysis (GSEA) revealed significant upregulation of pathways involved in ER homeostasis, including the UPR, protein processing in the ER, IRE1α/XBP1, and PERK signaling, in *Sel1L^MLC^* mice (Figure 7A). Downregulated pathways included genes involved in extracellular matrix and collagen organization (Supplemental Figure 5A). Among the top upregulated genes in *Sel1L^MLC^* muscle (red, Figure 7B and Supplemental Table 1) were fibroblast growth factor 21 (*Fgf21*), phosphoenolpyruvate carboxykinase 2 (*Pck2*), methylenetetrahydrofolate dehydrogenase 2 (*Mthfd2*), asparagine synthetase (*Asns*), cystine/glutamate transporter (*Slc7a11*), and phosphoserine aminotransferase (*Psat1*), which are known to be activated by the ISR/ATF4 pathway (41, 42), supporting our observations of enhanced ISR signaling.

Further analysis confirmed an approximately 70-fold increase in *Fgf21* mRNA and elevated FGF21 protein levels in *Sel1L^MLC^* muscle compared with WT littermates (Figure 7, C and D). Notably, FGF21 protein remained undetectable in the livers of the same mice under ad libitum conditions (Figure 7D), indicating that FGF21 induction was specific to muscle. FGF21 protein levels remained elevated in *Sel1L^MLC^* skeletal muscle between 4 weeks and 12 weeks of age (Figure 7E). Serum ELISA measurements revealed that both male and female *Sel1L^MLC^* mice exhibited a 30-fold increase in FGF21 levels under ad libitum conditions and even exceeded the levels observed in starved WT mice (Figure 7F), a physiological condition known to stimulate FGF21 (40, 41). Consistent with the reported effects of FGF21 on WAT (43), *Sel1L^MLC^* displayed significant beigeing in brown adipose tissue (BAT) and inguinal WAT of *Sel1L^MLC^* mice at 12 weeks of age, as evidenced by H&E staining and UCP1 expression (Figure 7, G and H, and Supplemental Figure 5B). A similar, albeit less extensive beigeing was observed at 4 weeks of age (Supplemental Figure 5C). These data suggest that SEL1L deletion in skeletal myocytes induces the expression of FGF21, leading to supraphysiological circulating FGF21 levels and the beigeing of adipose tissue.

### FGF21 links SEL1L-HRD1 ERAD in skeletal muscle to systemic metabolic regulation.

**Figure 8:**
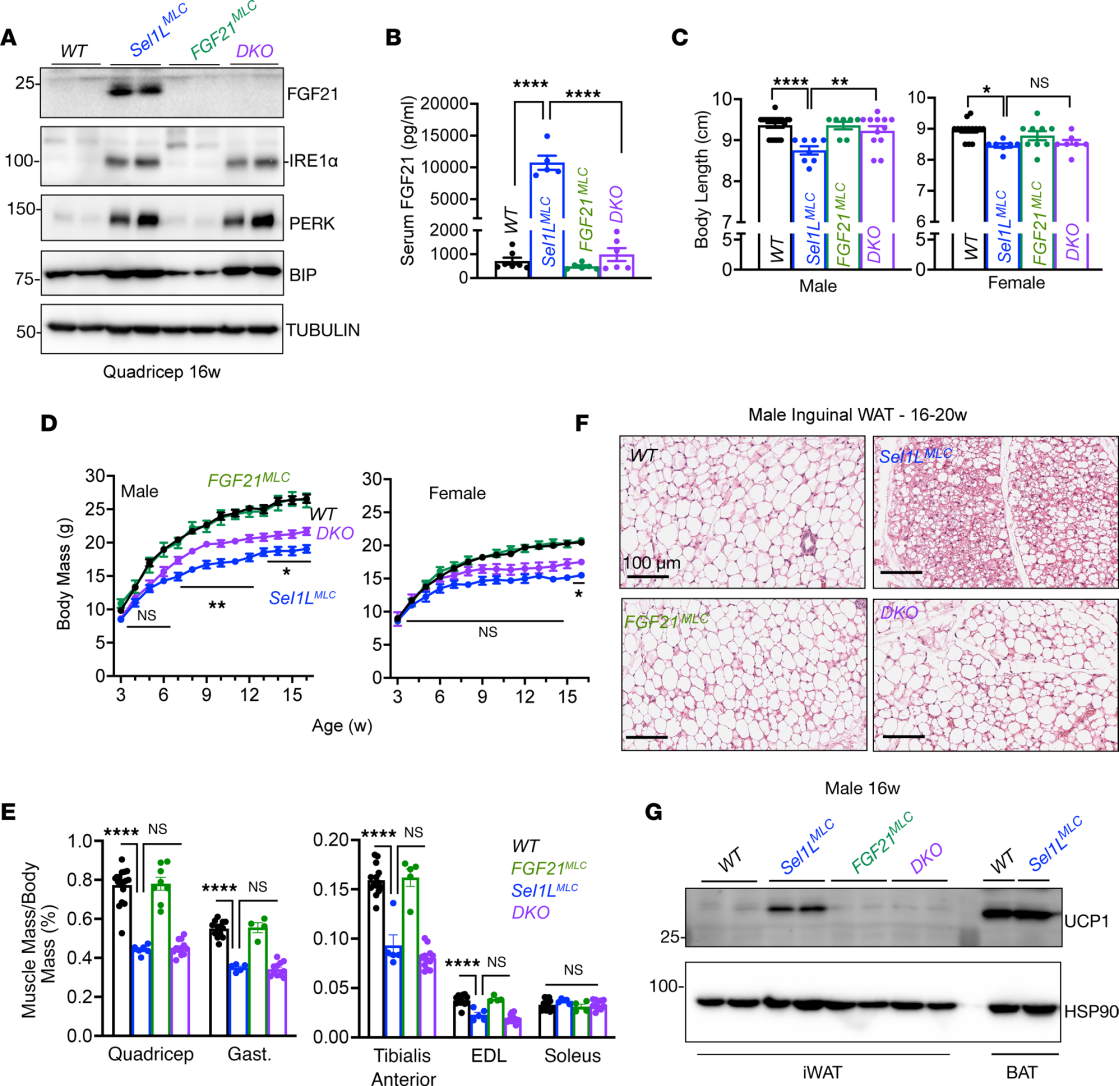
FGF21 links SEL1L-HRD1 ERAD in skeletal muscle to systemic metabolic regulation. (**A**) Representative Western blot of ER homeostasis proteins and FGF21 in WT, *Sel1L^MLC^* (SKO), *FGF21^MLC^* (FKO), and *Sel1L/Fgf21* double knockout (DKO) mice (*n* = 4 mice per genotype). (**B**) Serum measurement of FGF21 from male and female mice at 16–20 weeks old (*n* = 5–7 per genotype). (**C**) Body length of 16-week-old mice (*n* = 7–20 for males, *n* = 7–14 for females). (**D**) Weekly body mass of male and female mice (*n* = 6–44 per genotype/time point for males, *n* = 5–45 per genotype/time point for females). Statistical comparison made between *Sel1L^MLC^* and DKO mice. (**E**) Muscle mass–to–body mass ratios in male mice (*n* = 4–18 per genotype). Comparisons were made between indicated groups. FKO muscle was not statistically different from WT. (**F**) Representative H&E-stained images of inguinal white adipose tissue (*n* = 3 mice per genotype). (**G**) Representative Western blot of UCP1 in WT, *Sel1L^MLC^*, *FGF21^MLC^*, and DKO mice (*n* = 4 mice per genotype). Data presented as mean ± SEM. NS, *P* > 0.05; *P < 0.05; **P < 0.01; ****P < 0.0001 determined by 1-way ANOVA with Tukey’s multiple-comparison test (**B**, **C**, and **E**) or mixed-effects analysis (repeated-measure ANOVA) with Tukey’s multiple-comparison test (**D**).

To determine whether FGF21 contributes to the systemic changes in *Sel1L^MLC^* mice, we generated skeletal myocyte–specific SEL1L and FGF21 double knockout (DKO) mice. Western blot analysis revealed a near-complete loss of FGF21 protein level in the skeletal muscle of DKO mice, while ER markers such as IRE1α, PERK, and BiP were unchanged, compared to those in *Sel1L^MLC^* mice (Figure 8A). Circulating FGF21 levels in DKO mice were significantly reduced and comparable to WT levels (Figure 8B), providing definitive evidence for the source of circulating FGF21 levels in *Sel1L^MLC^* mice being in skeletal muscle. We further examined the impact of FGF21 deletion on organismal growth. While deletion of FGF21 in skeletal muscle alone (*Fgf21^MLC^*) had no effect on growth in both sexes, loss of FGF21 in DKO mice partially attenuated the growth restriction observed in *Sel1L^MLC^* mice (Figure 8, C and D). This effect was more pronounced in males than females, although the underlying reasons for this difference are currently unknown. Body length and body mass showed a mild rescue in DKO mice; however, impaired muscle growth persisted in DKO mice (Figure 8E), indicating that FGF21 was not responsible for the muscle growth impairment in *Sel1L^MLC^* mice. While the liver and adipose tissue mass were comparable between DKO and *Sel1L^MLC^* littermates (Supplemental Figure 6, A–C), FGF21 deletion in skeletal muscle attenuated the beigeing of iWAT and BAT as well as UCP1 expression in iWAT observed in *Sel1L^MLC^* mice (Figure 8, F and G, and Supplemental Figure 6D). These findings demonstrate that myocyte-derived FGF21 serves as the systemic signal that promotes adipose tissue beigeing in response to myocyte-specific SEL1L deficiency.

## Discussion

The ER is a versatile organelle responsible for protein synthesis, folding, and export of secretory proteins in skeletal muscle. However, our understanding of canonical ER function in skeletal muscle, particularly its regulatory control in muscle proteostasis, has been limited. In this study, we discovered that the SEL1L-HRD1 ERAD pathway in skeletal muscle, which plays a crucial role in managing misfolded proteins in the ER, is essential for muscle function and overall health. Our findings demonstrate that SEL1L-HRD1 ERAD acts as a primary regulator of ER homeostasis in muscle and is vital for promoting muscle hypertrophy during postnatal growth. Moreover, we reveal that ER protein management through SEL1L-HRD1 ERAD in myocytes is integrated with whole-body physiology. Loss of SEL1L in myocytes leads to reprogramming of systemic metabolism, including enhanced adipocyte beigeing, increased insulin sensitivity, and resistance to diet-induced obesity, partly through the action of myocyte-derived FGF21. These results unveil an unexpected physiological role of myocyte-specific SEL1L-HRD1 ERAD as a critical determinant of adult muscle mass, which is indispensable for systemic energy homeostasis.

Skeletal muscle is a highly adaptable organ that responds to a variety of physiological stimuli, including changes in diet and exercise. Additionally, pathophysiological conditions such as muscle injury or age-related muscle decline (sarcopenia) can disrupt proteostasis and trigger significant muscle loss (44, 45). These stimuli can lead to the accumulation of misfolded proteins, which must be promptly detected and cleared to prevent the formation of toxic aggregates that may impair muscle function (46, 47). The UPR is a cellular mechanism that mitigates ER misfolding by inducing the expression of genes involved in ER chaperones, promoting ER expansion, and enhancing ERAD. However, our study, along with 2 previous studies (48, 49), demonstrates that the UPR sensor IRE1α is dispensable for muscle growth under basal physiological conditions, indicating its limited role in muscle development. In contrast, we provide evidence that SEL1L-HRD1 ERAD is crucial for growing muscle (4 to 12 weeks of age), suggesting that the constitutive degradation of ER proteins through the SEL1L-HRD1 complex is necessary to maintain muscle growth. Given the increased rate of protein synthesis during muscle growth, numerous proteins originating from the ER and trafficked to the membrane, such as extracellular matrix components and sarcolemma proteins, may undergo rapid degradation via ERAD to balance protein load and ensure proteome fidelity. However, it remains to be determined whether ERAD exerts its effects on muscle growth by regulating specific substrates, similar to other mouse models deficient in SEL1L-HRD1 ERAD (11, 12), or whether it operates through a general mechanism to maintain proteostasis in a rapidly growing tissue.

Our observations indicate that myocyte-specific SEL1L deficiency robustly enhances various proteostatic pathways, such as mTOR signaling and autophagy, presumably as compensatory mechanisms in response to the loss of ERAD function. This suggests that the impact of ERAD deficiency extends beyond ER compartments and affects global proteostasis in muscle. This phenomenon may be attributed to the limited turnover of adult muscle cells and their restricted cytoplasm, which likely necessitate a robust and efficient system for protein clearance and management. The observed increase in autophagy in *Sel1L^MLC^* mice could be a mechanism to rapidly clear misfolded ER proteins in the absence of functional ERAD, preventing the formation of toxic aggregates that could compromise muscle contractility. Autophagy activation has been linked to increased UPR signaling (50); whether this occurs in SEL1L deficiency in muscle has yet to be determined. Providing support for this model, a recent study from our laboratory showed that IRE1α, an ERAD substrate, links ERAD deficiency to autophagy in pancreatic β cells (34), highlighting a potential interplay between ERAD, the UPR, and autophagy in vivo.

Accumulating evidence suggests that skeletal muscle senses local dysfunction and, in response, induces long-range (endocrine) adaptive changes to whole-body physiology (51–54). Although direct evidence is limited, we speculate that the increased energy expenditure and improved glucose metabolism observed in *Sel1L^MLC^* mice is a systemic adaptation in response to proteostatic/energy imbalance within the muscle. The enhanced glucose consumption in *Sel1L^MLC^* mice may be driven by the increase in beigeing of WAT and BAT, consistent with previous reports linking BAT function to improved metabolic parameters (55). The requirement for FGF21 in adipose tissue beigeing in *Sel1L^MLC^* mice supports the notion that FGF21 secretion coordinates systemic adaptation to muscle-intrinsic stress (51, 52, 56–61). However, it is important to note that FGF21 only partially rescued the body mass of *Sel1L^MLC^* mice and was dispensable for muscle growth, indicating that other factors likely contribute to the decreased size and altered metabolism observed in *Sel1L^MLC^* mice. These findings align with other muscle-specific manipulations that result in elevated FGF21 levels (such as mitochondrial dysfunction [UCP1-Tg] and autophagy [Atg7 deletion]), where ablation of FGF21 only partially attenuates some of the metabolic effects (51, 62). Thus, it is likely that the cell-autonomous effects impairing muscle growth in *Sel1L^MLC^* mice act independently of FGF21.

Activation of the ISR pathway has been associated with enhanced FGF21 production in skeletal muscle and is often linked to activated UPR and mitochondrial dysfunction (51, 52, 60, 61). Previous studies have shown that specific activation of the UPR sensor PERK in muscle is sufficient to enhance FGF21 secretion (59). Therefore, the elevated levels of PERK observed in *Sel1L^MLC^* mice could potentially contribute to increased ISR-mediated FGF21 production. Additionally, ISR-FGF21 activation is commonly associated with mitochondrial myopathy in both mice and humans (53, 58, 63). Loss of SEL1L has been found to impair mitochondrial function and dynamics in BAT (20). Furthermore, disrupted communication between the ER and mitochondria in muscle has been shown to impair muscle function (60). Thus, it is plausible that deficiency of SEL1L in skeletal muscle could also disrupt mitochondrial function and activate ISR signaling, which should be experimentally investigated in future studies. Overall, we speculate the loss of SEL1L may trigger a broad and overlapping network of stimuli, culminating in activation of the ISR/eIF2α/ATF4 axis in skeletal muscle. These processes, coupled with our observations of altered proteostasis, suggest that skeletal muscle may employ multiple strategies to compensate for the loss of ER protein degradation. While it is acknowledged that there may be additional mechanisms involved, our data support an integrated role of SEL1L-HRD1 ERAD that links ER protein degradation with systemic metabolism, shedding new light on how muscle copes with ER misfolding under physiological conditions.

## Methods

### Mouse strains and rearing conditions

All mouse strains used were on the C57BL/6J background, with both male and female sex used in experiments. Animal ages are reported in the figures and legends. Mice were fed on a standard lab diet (PicoLab Rodent Diet 5L0D) with free access to water, unless otherwise stated. Muscle-specific SEL1L (*Sel1L^MLC^*) and IRE1α (*Ire1α^MLC^*) knockout mice were generated by crossing *Sel1L^fl/fl^* mice (17) or *Ire1α^fl/fl^* (64) with mice expressing Cre driven by the myosin light chain promoter (MLC^Cre^); Cre-negative mice were used as controls. MLC^Cre^ mice were provided by Jiandie Lin at the University of Michigan, Ann Arbor, Michigan, USA. Muscle-specific Sel1L-FGF21 DKO littermate mice were generated by crossing heterozygous *Sel1L^MLC/+^* with *FGF21^fl/fl^* mice [Jackson Laboratory, stock 022361, B6.129S6 (SJL)-*Fgf21^tm1.2Djm^*/J], generating 4 cohorts of the following genotypes: *Sel1L^MLC^;FGF21^MLC/+^* (designated *Sel1L^MLC^*); *Sel1L^MLC/+^;FGF21^MLC^* (designated *Fgf21^MLC^*); *Sel1L^MLC^;FGF21^MLC^* (DKO); and WT mice (Cre negative). The phenotypes characterized in this study revealed that heterozygous mice were not different form WT mice in all genotypes used.

#### Mouse metabolic phenotyping.

All mice were housed in a temperature-controlled room with a 12-hour light/12-hour dark cycle and were fed normal chow diet (13% fat, 57% carbohydrate, and 30% protein, PicoLab Rodent Diet 5L0D). Indirect calorimetry was performed by the University of Michigan Animal Phenotyping Core using the comprehensive laboratory monitoring system (CLAMS, Columbus Instruments). Specifically, male mice were singly housed to acclimate for 3 days prior to being placed in CLAMS chambers. Oxygen consumption, carbon dioxide production, spontaneous motor activity, and food intake were continually monitored for 72 hours in an integrated open-circuit calorimeter equipped with an optical beam activity monitor. Body fat and lean mass were measured using an NMR analyzer (Minispec LF990II, Bruker Optics). The data reported in the manuscript have the first 24 hours of CLAMS analysis discarded for mouse acclimatization to metabolic cages.

#### HFD feeding.

Littermate mice undergoing HFD feeding (60% caloric intake from fat; Research Diets D12492) were group housed with mixed genotypes. Mice were continually fed with HFD for 12 weeks and monitored for body mass and health changes throughout the duration of feeding. Daily food intake was performed separately by first acclimating mice in single house cages for 3 days. HFD food pellets were then portioned and weighed into each cage daily, with the leftover food weighed and subtracted from the previous day. Measurements of food intake were taken for 7 consecutive days.

### Tissue and blood collection

For blood serum, animals were anesthetized with isoflurane followed by cardiac puncture and syringe extraction. Blood was kept at room temperature for 30 minutes followed by centrifugation at 3,000*g* for 15 minutes at 4°C, followed by storage at –80°C. Mice were then sacrificed by cervical dislocation and peripheral tissues (muscle, liver, fat) were harvested and snap-frozen in microfuge tubes placed in liquid nitrogen. Tissues were kept at –80°C until analysis for Western blotting or quantitative PCR. Blood lactate was measured via tail nick using a blood lactate meter (Lactate Plus) and Lactate Plus Meter Test Strips in rested, ad libitum–fed mice.

### Colchicine treatment

Colchicine treatments were adapted from Ju et al. (65), where 2 doses of colchicine (0.4 mg/kg) diluted in 1× PBS were administered at 12-hour intervals: one at 9 pm and again at 9 am on the following day via intraperitoneal injection. Control animals were administered an equivalent volume of 1× PBS via intraperitoneal injection. After treatments, animals were then sacrificed with isoflurane exposure followed by cardiac puncture and muscle tissues were collected. Autophagy flux was calculated by measuring the ratio of LC3-II (bottom band) to tubulin.

### Western blot analysis

Mouse tissues were minced and homogenized in microfuge tubes placed in ice. Approximately 50 mg of tissue was further homogenized in ice-cold lysis buffer (1% Triton X-100, 150 mM NaCl, 100 mM Tris-Cl pH 7.5, 1 mM EDTA, 1 mM EGTA) with protease inhibitor cocktail (Sigma-Aldrich) and phosphatase inhibitor (Sigma-Aldrich) until large pieces were not observed. Tissue lysates were incubated on ice for 30 minutes before being centrifuged at 4°C and 16,000*g* for 10 minutes. The supernatant was collected and analyzed for protein concentration assay using the Bradford assay (Bio-Rad). Depending on the antibody, the protein lysate was denatured at 65°C (10 minutes) or 95°C (5 minutes) in 5× SDS sample buffer (250 mM Tris-HCl pH 6.8, 10% SDS, 0.05% bromophenol blue, 50% glycerol, and 1.44 M β-mercaptoethanol). Approximately 30 to 40 μg of protein was loaded in each lane and resolved by SDS-PAGE, electrotransferred onto PVDF membranes, and probed with the following primary antibodies diluted in 2% BSA/TBST overnight at 4°C: anti-tubulin (Santa Cruz Biotechnology, sc-5286; 1:5000), anti-HSP90 (Santa Cruz Biotechnology, sc-13119; 1:5000), anti-SEL1L (made in house, ref. 20; 1:10,000); anti-HRD1 (Proteintech, 13473-1-AP; 1:5000), anti-BiP (Abcam, ab21685; 1:1000), anti-calnexin (Enzo Life Sciences, ADI-SPA-860; 1:1000), anti-PDI (Enzo Life Sciences, ADI-SPA-890; 1:1000), anti–p70-S6K (Santa Cruz Biotechnology, sc-8418; 1:000), anti–p70-S6K^Ser434^ (Santa Cruz Biotechnology, sc-8416; 1:1000), anti-AKT (Cell Signaling Technology, 9272; 1:1000), anti–p-AKT^Ser473^ (Cell Signaling Technology, 9271; 1:1000), anti-4EBP1 (Cell Signaling Technology, 9644; 1:1000), anti–p-4EBP1^Ser65^ (Cell Signaling Technology, 9451; 1:1000), anti LC3 (Cell Signaling Technology, 2775; 1:1000), anti–caspase-3 (Cell Signaling Technology, 9662; 1:1000), anti–cleaved caspase-3 (Cell Signaling Technology, 9661; 1:1000), anti-IRE1α (Cell Signaling Technology, 3294; 1:1000), anti-PERK (Cell Signaling Technology, 3192; 1:1000), anti-FGF21 (R&D Systems, AF3057; 1:500), anti-EIF2α (Cell Signaling Technology, 9722; 1:1000), anti–p-EIF2a^Ser51^ (Cell Signaling Technology, 9721; 1:1000), anti-ATF4 (Cell Signaling Technology, 11815; 1:1000), and anti-UCP1 (Proteintech, 23673-1-AP; 1:1000). Membranes were washed 3 times with 1× TBST and incubated with secondary antibodies (diluted 1:5000 in 5% milk, for blocking) either goat anti–rabbit IgG HRP, goat anti–mouse IgG HRP, or donkey anti-goat HRP, at room temperature for 1 hour. Membranes were washed an additional 3 times in 1× TBST and imaged using ECL reagents and a Bio-Rad ChemiDoc System. Band intensities were determined using Bio-Rad Image Lab software. See complete unedited blots in the supplemental material.

### Phosphatase treatment

Phosphatase treatment was performed following the manufacturer’s protocol. Briefly, 40 μg of tissue lysate sample was incubated with 1 μL of λ-phosphatase (New England Biolabs) in 1× NEBuffer and incubated at 30°C for 30 minutes. Liver tissue extracted from mice 24 hours after a single intraperitoneal injection with the ER stress inducer tunicamycin (1 mg/kg) was used as a positive control.

### Puromycin and protein synthesis

The puromycin procedure was adapted from Goodman and Hornberger (66) using the SUnSET assay. Briefly, mice were isoflurane anesthetized and injected intraperitoneally with 0.04 μM/g body mass of puromycin (Sigma-Aldrich). After 30 minutes, mice were sacrificed and hindlimb muscles and liver were collected and snap-frozen in liquid nitrogen. Actively synthesized proteins were detected with anti-puromycin (EMD Millipore, clone 12D10; 1:2500 dilution). Densitometry analysis of full individual lanes was normalized to total protein (Ponceau S staining).

### RNA extraction for qPCR and microarray.

Total RNA from tissues was extracted using either TRIzol or an RNeasy Micro Kit (QIAGEN). RNA concentration was determined using the NanoDROP 2000 UV-Vis Spectrophotometer. RNA quality and concentration for the microarray was determined using the RNA 6000 Nano Kit on an Agilent 2100 bioanalyzer. One microgram of RNA was used to generate cDNA, with quantitative real-time PCR amplification performed on an Applied Biosystems QuantStudio 5. Relative expression was normalized to reference genes *Actin* or *L32*. PCR primers and experimental conditions are described in Supplemental Table 2. The microarray was performed as previously described (27). To generate the volcano plot, genes were sorted by fold change in *Sel1L^MLC^* expression over WT and plotted against *P* values as determined by intensity-based moderated T-statistic (IBMT).

### Histology and immunofluorescence

For H&E staining, nonmuscle tissue (BAT, WAT, liver, pancreas) was dissected and fixed in 10% formalin overnight at 4°C. Tissue samples were embedded in paraffin, cut, and stained with H&E by the University of Michigan Rogel Cancer Center Tissue and Molecular Pathology Core. For muscle H&E staining, the tibialis anterior muscles were dissected and immediately frozen in isopentane cooled by liquid nitrogen. Muscles were embedded in OCT, sectioned with a cryostat at 10 μm slices at the mid-belly, and mounted on microscope slides at room temperature. Microscope slides were kept at –80°C until further processing. A standard H&E staining protocol was performed on muscle frozen sections and all tissues were imaged and scanned using an Aperio Scanscope (Leica Biosystems). For immunofluorescence of isolated myofibers, freshly dissected EDL muscles were fixed in 4% paraformaldehyde overnight at 4°C. Fixed muscle bundles were then carefully teased apart using fine forceps prior to permeabilization and staining steps. Both frozen sections (on slides) and isolated myofibers (free floating) followed the same staining protocol. Muscle samples (on slides) or isolated muscle fibers (in 1.5 mL microcentrifuge tubes) were permeabilized in 0.2% Triton X-100 for 10 minutes. Muscle tissue was washed 3 times in PBS for 5 minutes at room temperature followed by a blocking buffer (5% normal donkey serum/1× PBS) for 60 minutes in a humidified chamber. Primary antibodies were diluted in antibody dilution buffer (0.1% Triton X-100 buffer/1% BSA) in a humidified chamber at 4°C overnight. The following antibodies were used: anti-SEL1L (made in house; 1:1000), anti-KDEL (Novus, NBP1-97469; 1:500), anti-laminin (Abcam, ab11575; 1:200), anti-RYR1 (Proteintech, 66539; 1:500), anti-dystrophin (Sigma-Aldrich, D8168; 1:200), and anti-BiP (Abcam, ab21685; 1:500). The next day, tissue was washed 3 times for 10 minutes each in antibody dilution buffer, followed by incubation with secondary antibodies (Alexa Fluor 488 and/or Alexa Fluor 647) for 1 hour in the dark at room temperature. Tissue was washed 3 more times in antibody dilution buffer and counterstained with the Vectashield mounting media containing DAPI (Vector Laboratories). Confocal images were acquired using the Nikon A1 confocal laser microscope (Michigan Diabetes Research Center Microscopy, Imaging, and Cellular Physiology Core). Image analysis was performed on NIH ImageJ (FIJI).

To assess muscle fiber type composition, tibialis anterior muscle cryosections on microscope slides were incubated with a 1:300 dilution of antibodies against the following MHC subtypes purchased from Developmental Studies Hybridoma Bank; type I (clone BA-F8), MHC type IIa (clone 2F7), type IIb (clone 10F5), and rabbit anti-laminin (Abcam, ab11575; 1:200) used to stain muscle membranes. After overnight incubation and PBS washes, muscle sections were incubated for 1 hour at room temperature with the following secondary antibodies at 1:500 dilution (Thermo Fischer Scientific, Invitrogen); Alexa Fluor 568 goat anti-mouse IgG1, Alexa Fluor 647 goat anti-mouse IgG2b, Alexa Fluor 488 goat anti-mouse IgM, and Alexa Fluor 647 goat anti-rabbit IgG. Slides were washed 3 times with PBS, mounted with Vectashield, and imaged using a Nikon A1 confocal microscope. Fiber composition was assessed with NIH ImageJ (FIJI), with type IIx fibers classified as unstained (black) fibers.

### Morphometric analysis

Whole tibialis anterior muscle H&E-stained cross sections were digitally scanned and imaged using an Aperio Scanscope. Images were then analyzed by NIH ImageJ (FIJI) software where approximately 100 to 200 myofibers per mouse were manually calculated for individual cross-sectional area. Due to the significant size difference between *Sel1L^MLC^* and WT muscle, relative fiber number was determined in a blind analysis, by calculating whole muscle tissue cross-sectional area, and then counting all myofibers within a 25% section of each individual muscle. For adipocyte size, H&E-stained sections of gonadal WAT were scanned and imaged at 10× magnification and cross-sectional area was calculated for approximately 150 to 200 adipocytes per animal using NIH ImageJ (FIJI) software. Similarly, ER/SR membranes in the EDL muscles were manually calculated from transmission electron microscopy images taken at the A-I junction in the muscle fiber. Images were acquired from multiple individual fibers from 3 mice per genotype.

### Transmission electron microscopy

Mice were anesthetized and perfused with cold PBS followed by fixation buffer (3% formaldehyde, 3% glutaraldehyde, in cacodylate; Electron Microscopy Sciences). The EDL and tibialis anterior muscles were dissected and fixed overnight at 4°C in 3% glutaraldehyde, 3% formaldehyde in 0.1 M Sorenson’s buffer (Electron Microscopy Sciences). The muscle tissue was prepared, embedded, and sectioned either longitudinally or transversally by the University of Michigan Biomedical Research Core Facilities. High-resolution images were acquired with a JEOL 1400-plus electron microscope.

### Serum measurements

FGF21 serum analysis was performed using a FGF21 Mouse ELISA kit (BioVender), following the manufacturer’s protocol. Serum insulin analysis was performed using a mouse ELISA kit (Crystal Chem, 90080), following the manufacturer’s protocol. Random blood glucose was taken via tail nick using a glucometer and OneTouch Ultra Test Strips.

### GTT and ITT

Mice were fasted 5–6 hours before GTT and ITT procedures and basal blood glucose was measured using a standard glucometer and One-Touch Ultra glucose strips. For the GTT procedure, fasted mice were intraperitoneal injected with a 1.5 g/kg body mass bolus of glucose and blood glucose was measured via tail nick at 15, 30, 60, and 90 minutes. For the ITT procedure, fasted mice were intraperitoneally injected with 0.75 U/kg body mass of recombinant human insulin (Novolin R; Novo Nordisk) and blood glucose was measured at 10, 20, 30, and 60 minutes. Mice that fell below the detection limit of the blood glucose monitor were plotted at 30 mg/dL.

### Statistics

All data are reported as mean ± SEM and statistical analyses were performed in Prism 9 (GraphPad Software). All experiments were repeated twice or had at least 3 individual biological repeats. Unless otherwise stated, comparisons between 2 groups were analyzed using 2-tailed, unpaired *t* test. Comparison between 2 or more groups were made using 1-way or 2-way ANOVA. Weekly body mass analysis was performed using mixed-effect analysis (repeated-measure ANOVA). ANCOVA was performed as described by the NIDDK mouse metabolic phenotyping centers (MMPC, www.mmpc.org). *P* values less than 0.05 were considered statistically significant.

### Study approval

Animal work was formed in accordance with University of Michigan Institutional Animal Care and Use Committee at the University of Michigan Medical School (PRO00010658)

### Data availability

All data and materials for the manuscript are included in the Methods and Supporting Data Values. Materials and reagents are freely available upon request. Microarray data are deposited in the NCBI Gene Expression Omnibus database (GEO GSE237194).

## Author contributions

BA designed and performed most of experiments. Y Liang assisted with experiments. MT processed and imaged the TEM samples. NS and RBR performed insulin and glucose experiments. DLS, ABL, and BP performed some muscle/histological analysis. Y Lu assisted with bioinformatic analysis. SK performed microarray analysis. LQ directed the project. BA and LQ conceived the project and wrote the manuscript. All other authors edited and approved the manuscript.

## Supplementary Material

Supplemental data

Supporting data values

## Version 1 08/03/2023

In-Press Preview

## Version 2 09/08/2023

Electronic publication
