# Supplementary material for: Muscle-specific ER-associated degradation maintains postnatal muscle hypertrophy and systemic energy metabolism
Source: JCI Insight. 2023 Sep 8;8(17):e170387. doi: 10.1172/jci.insight.170387 (PMC10578429; doi:10.1172/jci.insight.170387)

SUPPLEMENTAL MATERIALS FOR

Muscle specific ER-associated degradation maintains postnatal muscle hypertrophy and systemic energy metabolism

Benedict Abdon[1], Yusheng Liang[1,6], Débora da Luz Scheffer[1,2] Mauricio Torres[1], Neha Shrestha[1], Rachel B. Reinert[4], You Lu[1], Brent Pederson[1], Amara Bugarin-Lapuz[1] , Sander Kersten[3] and Ling Qi[1,4,5,6*]

SUPPLEMENTAL FIGURES AND LEGENDS

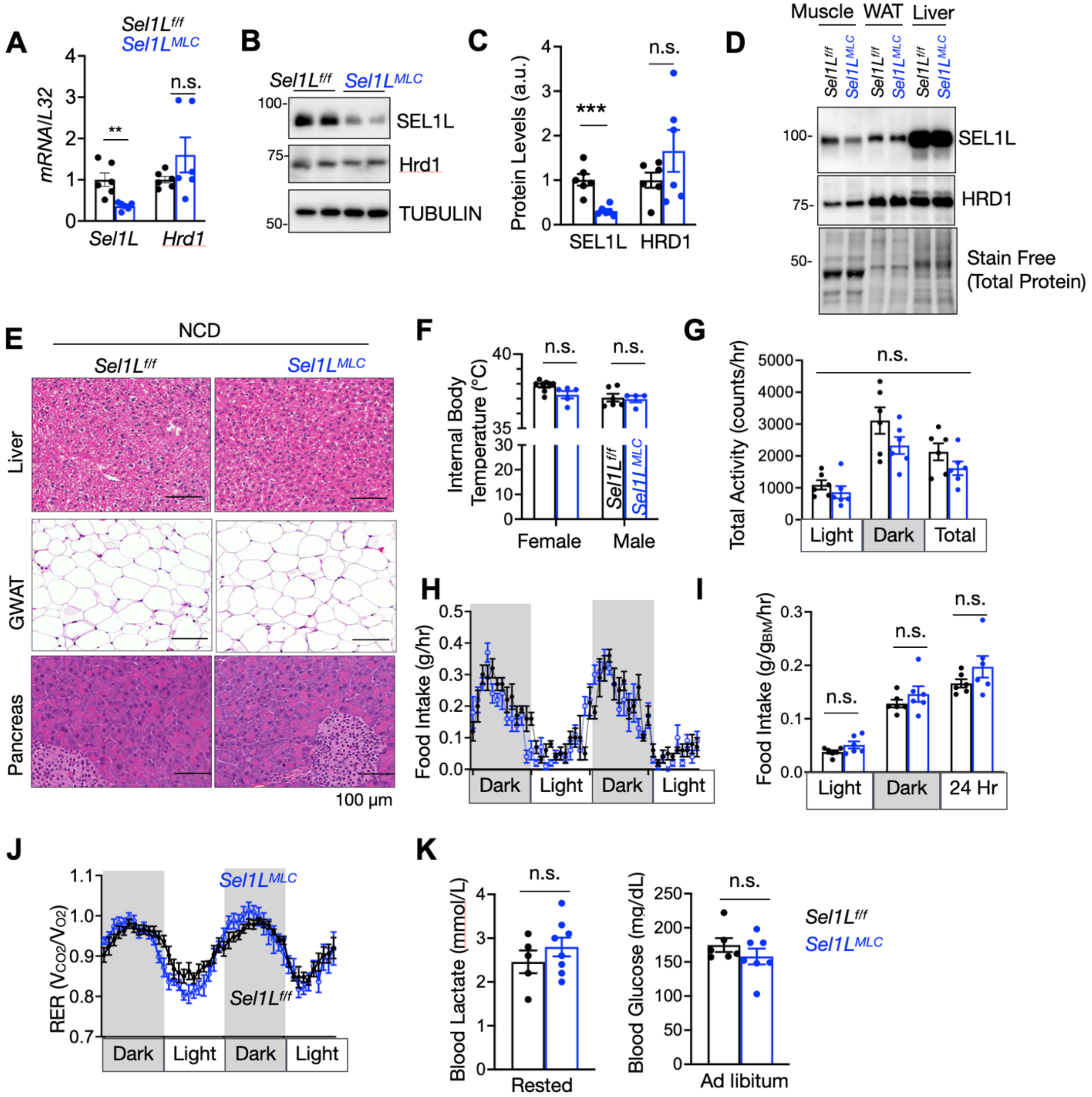

Supplemental Figure 1. Generation and characterization of *Sel1L*^{MLC} mice under basal conditions. (**A**) qPCR of *Sel1L* and *Hrd1* genes in quadricep muscle in 8-week old mice (n=6 mice per genotype). (**B**) Representative western blot of muscle and (**C**) Quantitation of protein levels of SEL1L and HRD1 in muscle (n=6 per genotype). (**D**) Representative western blot of SEL1L and HRD1 in muscle, white adipose tissue and liver (n=3 mice per genotype). (**E**) Representative H&E stain of liver, gonadal WAT, and pancreas from 12 week old mice (n=5 mice per genotype). (**F**) Internal body temperature of male and female mice (n=4-7 mice per genotype/sex). (**G**) Total activity (locomotor) of 10 week old male mice (n=6 mice per genotype). (**H**) Absolute food intake of 10 week old male *Sel1L*$^{f/f}$ and *Sel1L*^{MLC} mice and (**I**) Food intake normalized to lean body mass of 10 week old male mice (n=6 mice per genotype). (**J**) Respiratory exchange ratio (RER) of 10 -week-old male mice (n=6 mice per genotype). (**K**)

Blood lactate and glucose at basal resting conditions (n=5-8 per genotype). Data, mean ± SEM; n.s., $p > 0.05$; *, $p<0.05$; **, $p < 0.01$; *** $p < 0.001$ determined by two-tailed unpaired t-test (A, C, F, G, I, K).

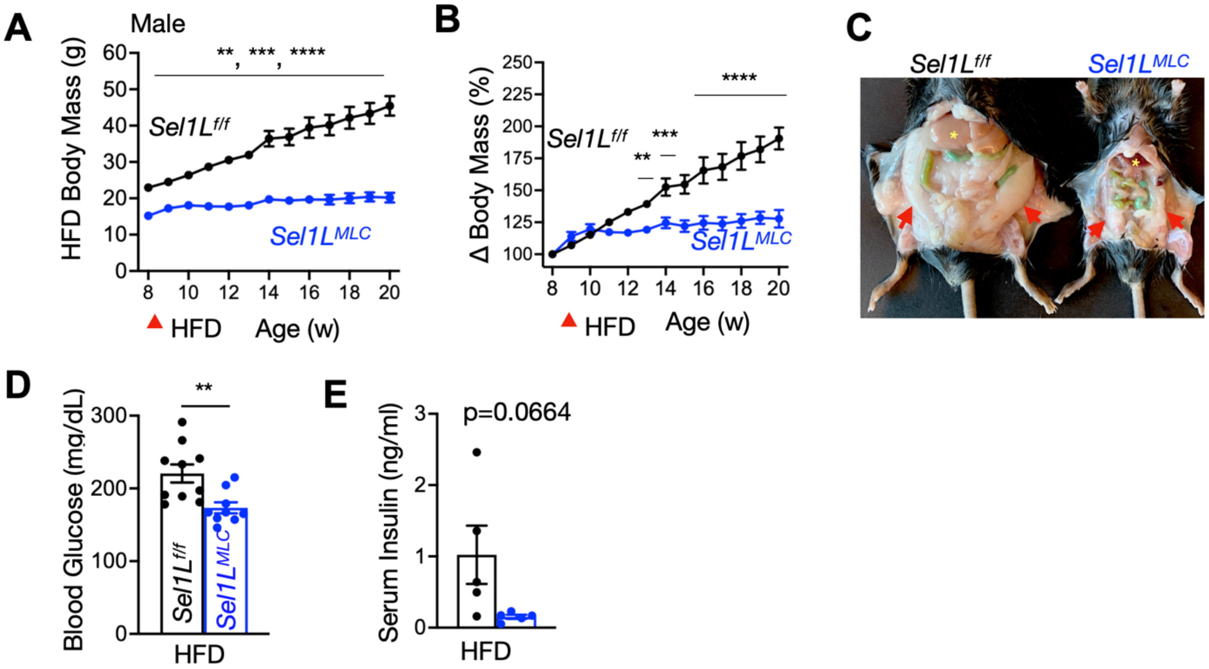

Supplemental Figure 2. *Sel1L^{MLC}* mice are resistant to diet-induced obesity.
(**A**) Body mass curve of male mice during 12 weeks of high fat diet (n=4-11 mice per genotype/time point). Red arrow indicates the start of high fat diet feeding (Day 0)**.** (**B**) Body mass (percentage) relative to Day 0 during high fat diet feeding (n=4-11 mice per genotype/time point). Red arrow indicates the start of high fat diet feeding (Day 0) (**C**) Representative photograph of male mice after 12 weeks of high fat diet (**D**) Blood glucose of male mice under high fat diet. (n=9-10 mice per genotype). (**E**) Blood serum insulin of male mice under high fat diet (n=5 mice per genotype). Data, mean ± SEM; n.s., p > 0.05; *, p<0.05; **, p < 0.01; *** p < 0.001 determined by Mixed-effects analysis (Repeated Measures) with Tukey's multiple comparisons test (A, B), or two-tailed unpaired-t-test (D,E)

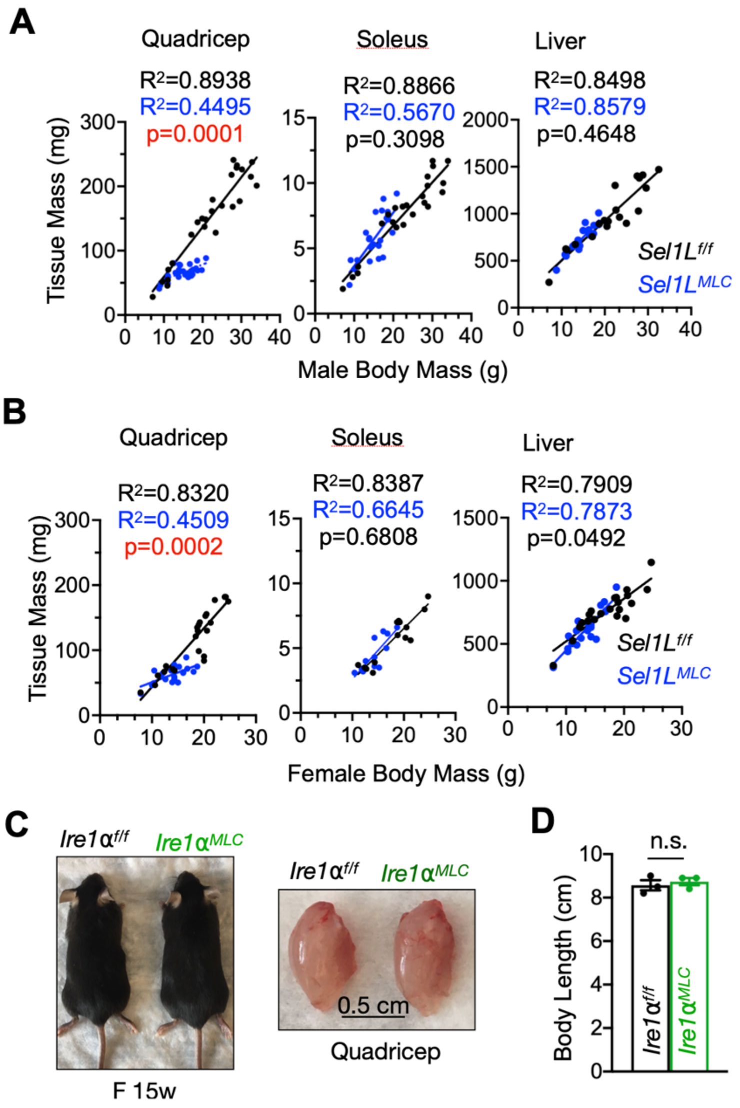

Supplemental Figure 3. Postnatal hypertrophic growth of skeletal muscle requires SEL1L-HRD1 ERAD, but not IRE1α.

(**A**) Organ mass to body mass plot of male mice aged 4-20 weeks old. Each point indicates one mouse (n=15-19 mice per genotype/tissue) (**B**) Organ mass to body mass plot of female mice aged 4-20 weeks old. Each point indicates one mouse. (n=12-21 mice per genotype/tissue). (**C**) Representative photo of *Ire1α*$^{f/f}$ and *Ire1α*^{MLC} mice and quadricep muscle. (**D**) Body length measurement of 12-week-old *Ire1α*$^{f/f}$ and *Ire1α*^{MLC} mice (n=3 mice per genotype). Data, mean ± SEM; n.s., $p > 0.05$ determined by two-tailed unpaired-t-test (D). r^2 values represent goodness of fit, p-value represents difference in slope between genotypes as determined by simple linear regression analysis (A,B).

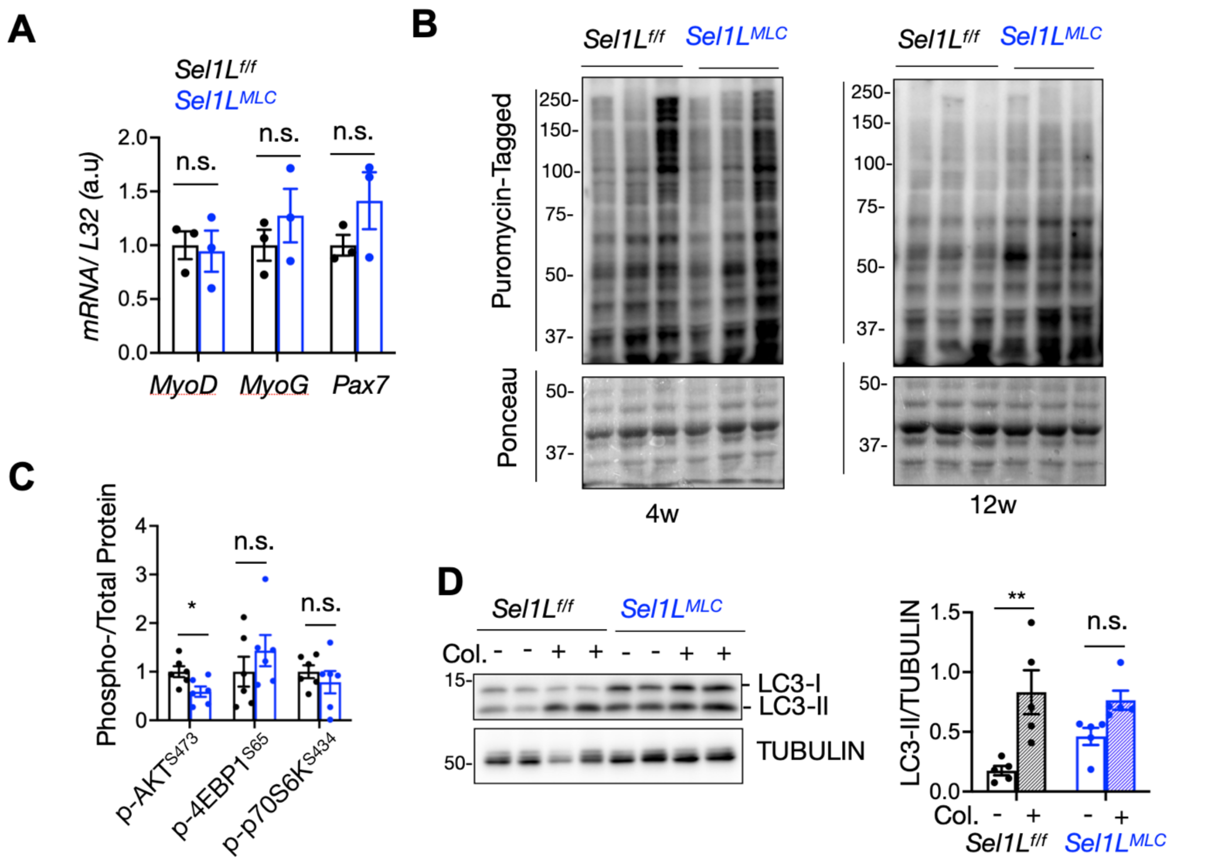

Supplemental Figure 4. SEL1L deficiency in muscle alters global proteostasis. (**A**) qPCR of myogenic genes *MyoD*, *MyoG*, *Pax7* in 8-week-old muscle (n=3 mice per genotype). (**B**) Representative western blot of puromycin-tagged proteins in 4- and 12-week-old muscles (n=3-5 mice per genotype). (**C**) Quantitation of Figure 4B (n=6 mice per genotype) (**D**)Representative western blot and quantitation of LC3 conversion after colchicine (0.4mg/kg/day, for 2 days) treatment (n=5 mice per genotype/treatment). Data mean ± SEM; n.s., p > 0.05; *, p<0.05; **, p < 0.01; *** p < 0.001; **** p < 0.001 determined by two-tailed unpaired t-test (A, C) or Two-way ANOVA with Tukey's multiple comparison's test (D).

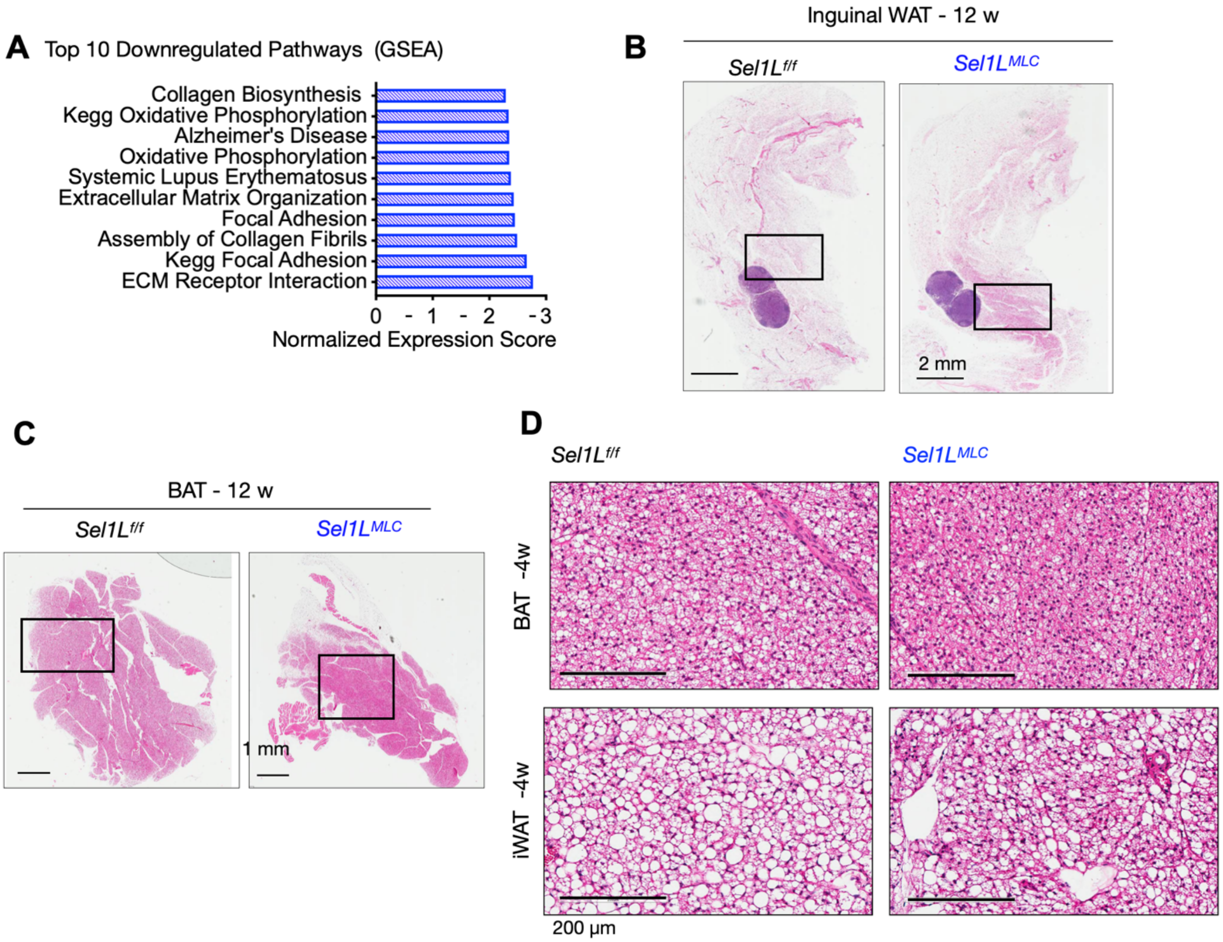

Supplemental Figure 5. Increased BAT and iWAT beiging in *Sel1L*^{MLC} mice.
(**A**) Gene set enrichment analysis (GSEA) of downregulated pathways in female 8-week old gastrocnemius muscle (n=3 mice per genotype). (**B**-**C**) Low magnification of H&E staining in iWAT and BAT from Fig 7G, region of interest indicated by black box (n=3 mice per genotype). (**C**) Representative H&E stain of brown and inguinal white adipose tissue from 4 weeks old male mice (n=3 mice per genotype).

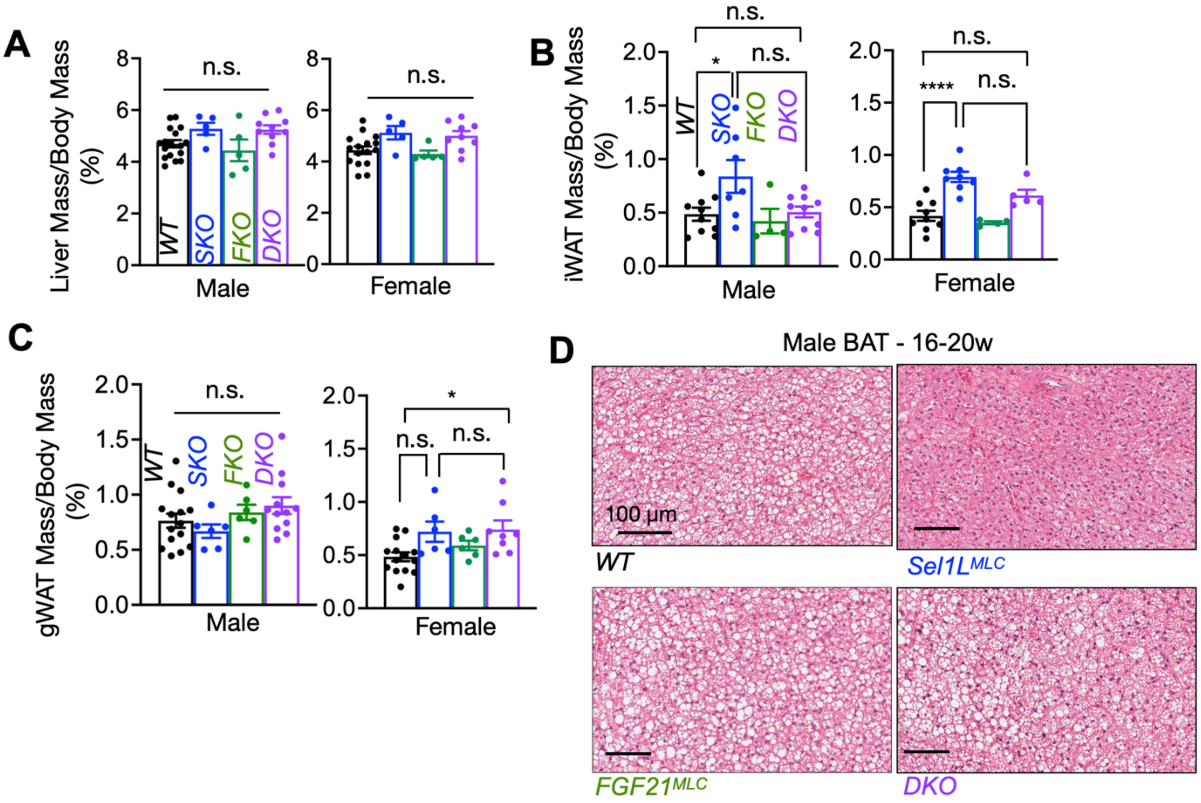

Supplemental Figure 6. Muscle-specific *FGF21* deletion does not affect muscle, liver, or adipose tissue growth in *Sel1L*^{MLC} mice. (**A**) Liver mass normalized to body mass in 16-20 week-old *WT*, *Sel1L*^{MLC} *(SKO)*, *FGF21*^{MLC} *(FKO)*, and *DKO* mice (n=5-17 per genotype/sex). (**B**) Inguinal white adipose tissue mass (iWAT) normalized to body mass in 16-20 week-old *WT*, *Sel1L*^{MLC} *(SKO)*, *FGF21*^{MLC} *(FKO)*, and *DKO* mice (n=4-13 mice per genotype/sex. (**C**) Gonadal white adipose tissue mass normalized to body mass 16-20 week-old *WT*, *Sel1L*^{MLC} *(SKO)*, *FGF21*^{MLC} *(FKO)*, and *DKO* mice (n=6-15 mice per genotype/sex. (**D**) Representative H&E images of brown adipose tissue (n=3 mice per genotype). Data mean ± SEM; n.s., p > 0.05; *, p<0.05; **, p < 0.01; *** p < 0.001; **** p < 0.001 determined by One-way ANOVA with Turkey's multiple comparisons test. Comparison of tissue mass between WT and FKO was not statistically different in all measurements.

Supplemental Table 1

Top 20 Upregulated Genes		
**Gene Name**	**Fold Change (KO/WT)**	**Full Name**
Fgf21	27.2	Fibroblast Growth Factor 21
Pck2	14.76	Phosphoenolpyruvate Carboxykinase 2 (mitochondrial)
Mthfd2	14.2	Methylenetetrahydrofolate Dehydrogenase
Asns	11.6	Asparagine Synthetase
Bora	9.45	BORA Aurora Kinase A activator
Vdr	9.11	Vitamin D Receptor
Sln	8.11	Sarcolipin
Psat1	8.01	Phosphoserine Aminotransferase 1
Slc17a9	7.4	Solute Carrier Family 17, member 9
D10Bwg137 (Arfgeg3)	7.09	ARGEF Family Member 3
Slc7a11	6.48	Solute Carrier family 7, member 11 (xCT)
Cdsn	6.41	Corneodesmosin
Naprt	6.31	Nicotinate Phosphoribosyltransferase
Mageh1	5.58	Melanoma Antigen, Family H, 1
Col20a1	5.54	Collagen, Type XX, Alpha 1
Aldh18a1	5.48	Aldehyde Dehydrogenase 18 family, member A1
Pgm3	5.46	Phosphoglucomutase 3
Uba5	5.4	Ubiquitin-like Modifier Activating Enzyme 5
Creb3	5.31	cAMP Responsive Element Binding Protein 3

Supplemental Table 2

RT-PCR Primers		
**Mouse Gene**	**Forward**	**Reverse**
Sel1L	CTGACTGAGGAAGGGTCTC	GCTAAAAACATTACAAAGGGGCA
Fgf21	GGGCTCTGATAAAGCATTCC	CAGCACTAAGGGAGGCAGAG
Ire1α	CCGAGCCATGAGAAACAAGG	CCCTGCCAGGATGGTCATGG
Cre	AGCGATGGATTTCCGTCTCT	CACCAGCTTGCATGATCTCC
Xbp1	ACGAGGTTCCAGAGGTGGAG	AAGAGGCAACAGTGTCAGAG
L32	GAGCAACAAGAAAACCAAGCA	TGCACACAAGCCATCTACTCA
**qPCR Primers**		
Sel1L	AGCCAGTCTTGACTGCCATT	ACCACAGTCTGCCATCTTCC
Hrd1	AGCTACTTCAGTGAACCCCACT	CTCCTCTACAATGCCCACTGAC
MyoD	CCCCGGCGGCAGAATGGCTACG	GGTCTGGGTTCCCTGTTCTGTGT
MyoG	GCAATGCACTGGAGTTCG	ACGATGGACGTAAGGGAGTG
Ire1α	ATCTGCGCAAATTCAGAACC	CTCCATGGCTTGGTAGGTGT
Perk	TCAAGTTTCCTCTACTGTTCACTCA	TTTGTTCCAGATGAACTCATGG
BiP	TGTGGTACCCACCAAGAAGTC	TTCAGCTGTCACTCGGAGAAT
Pax7	CTCAGTGAGTTCGATTAGCCG	AGACGGTTCCCTTTGTCGC
Fgf21	CTGGGGGTCTACCAAGCATA	CACCCAGGATTTGAATGACC
Actin	TCGTTGCCGGTCCACACCCG	CTCCTCAGGGGCCACACGCAG

Full Unedited Gels

Figure 1A

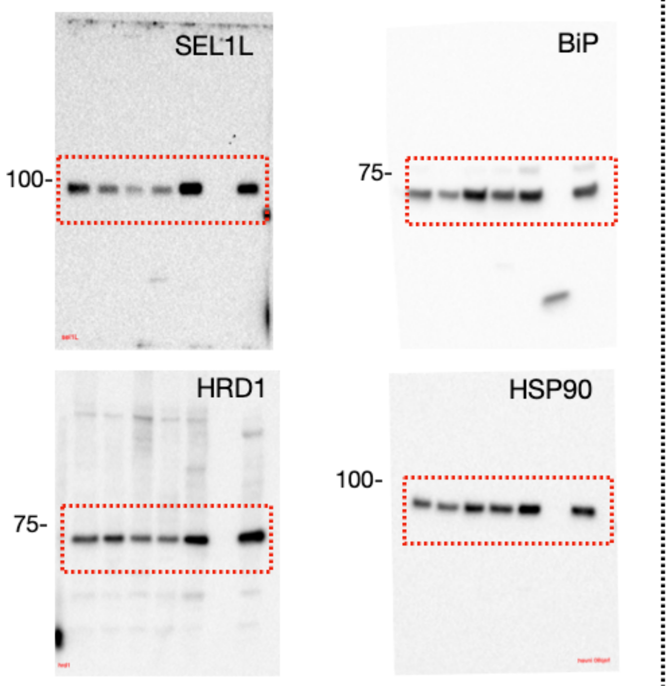

Figure 1E

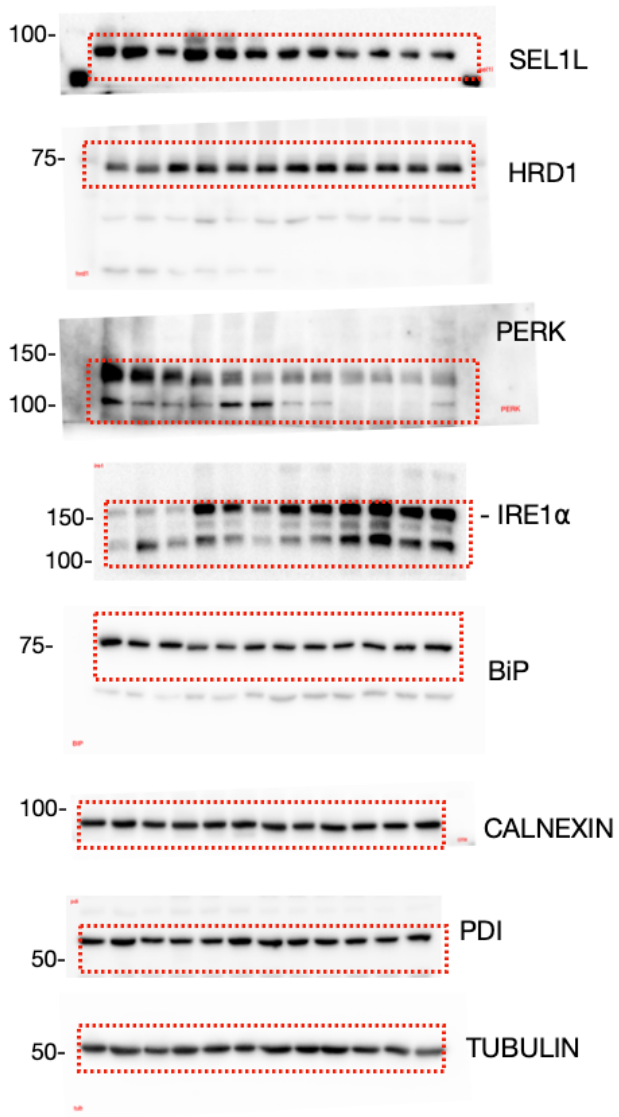

Sup. Figure 1B

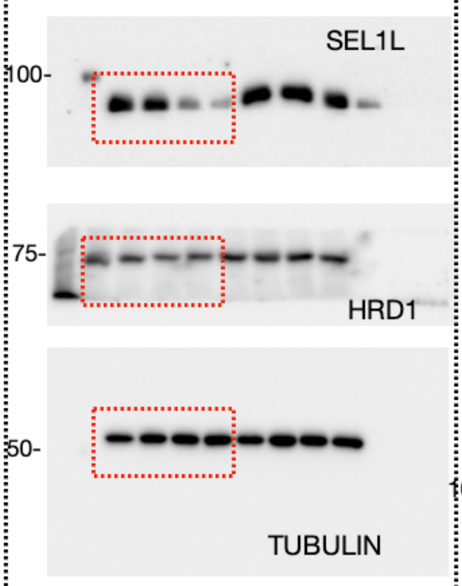

Sup. Figure 1D

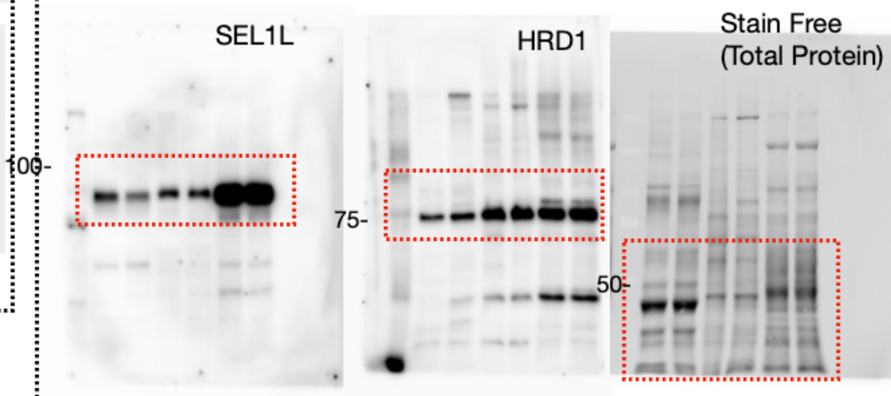

Figure 4B

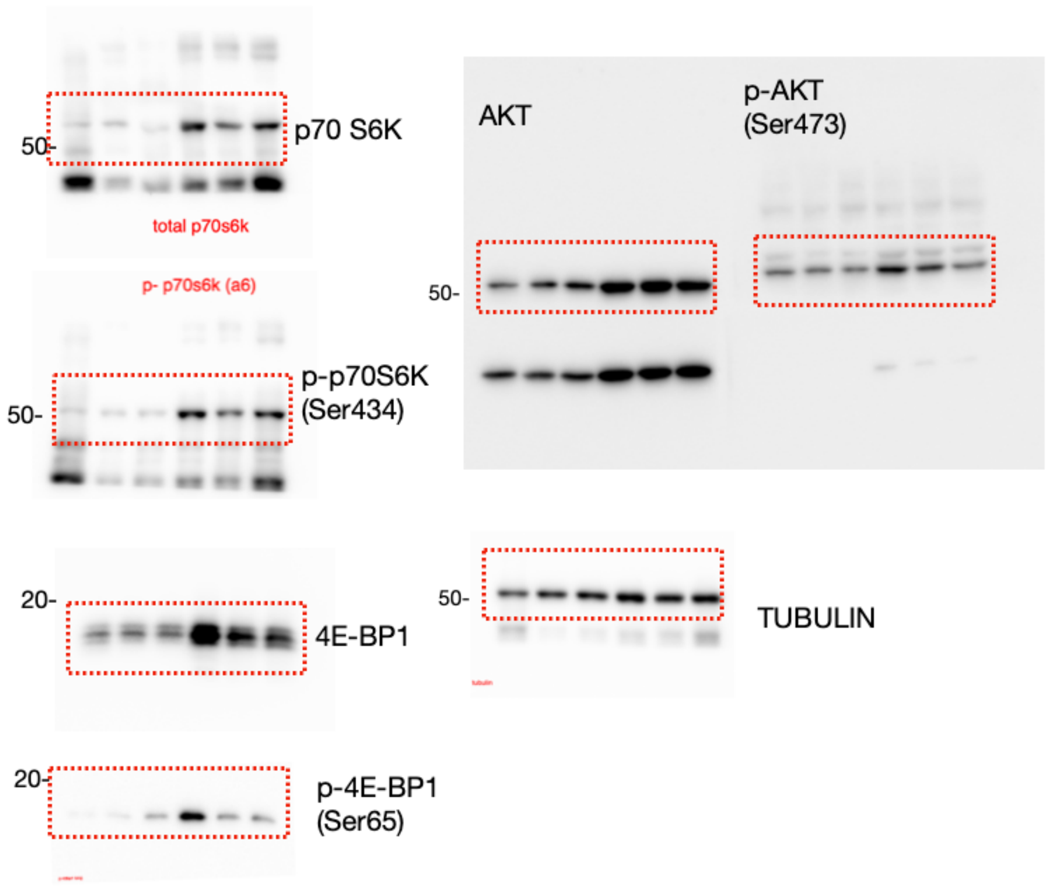

Figure 4D

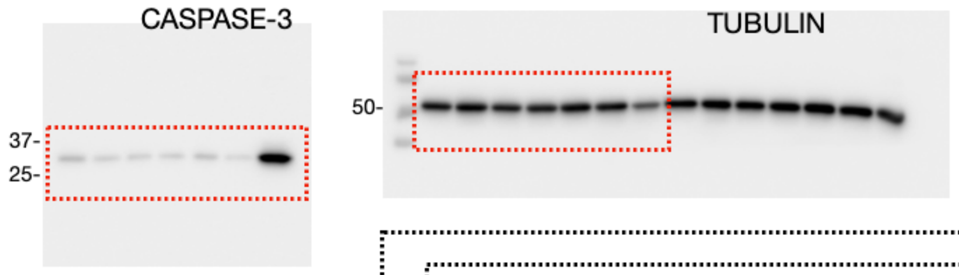

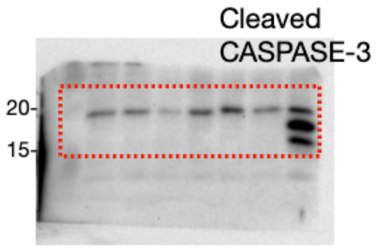

Figure 4E

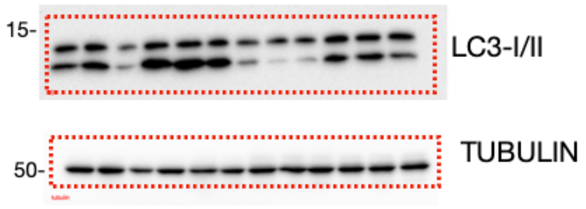

Sup. Figure 4B

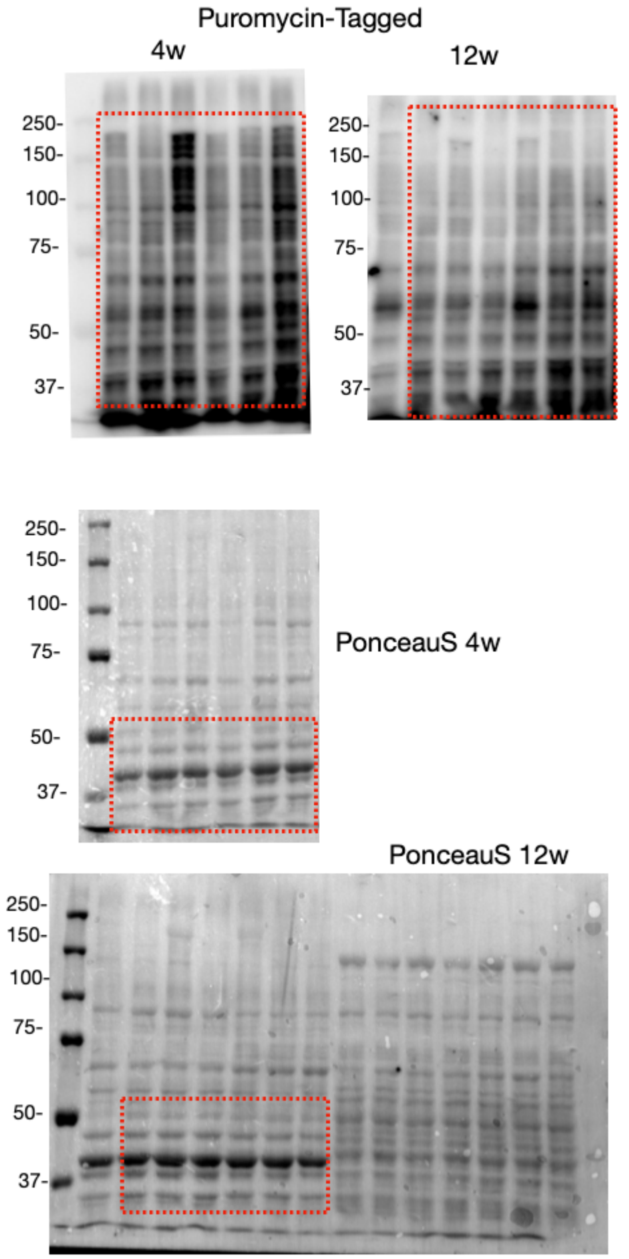

Figure 5C

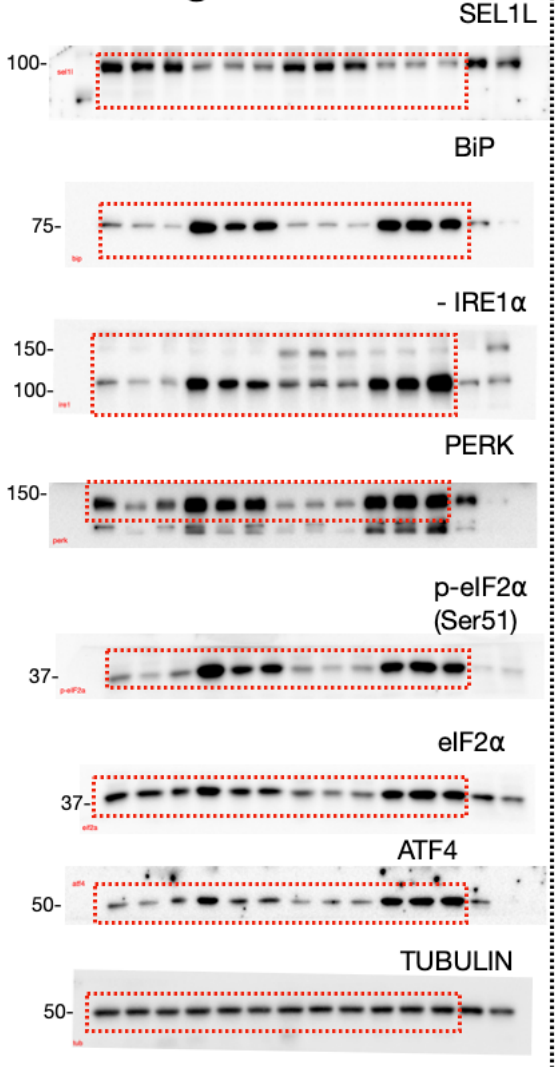

Figure 5H

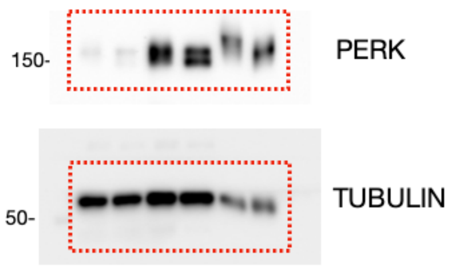

Figure 4D

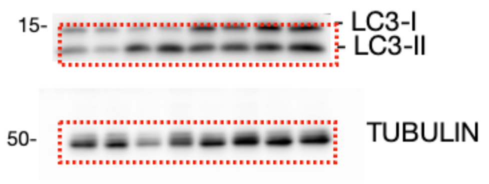

Figure 5G RT-PCR

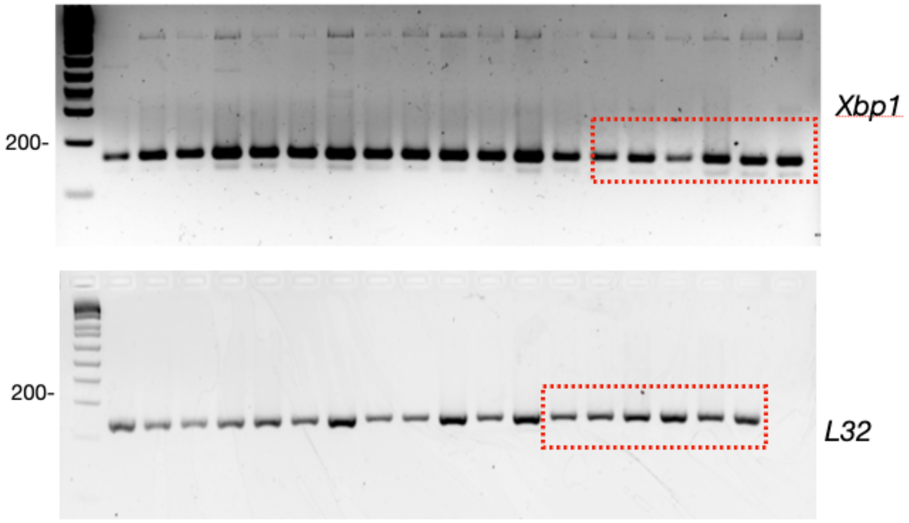

Figure 7D

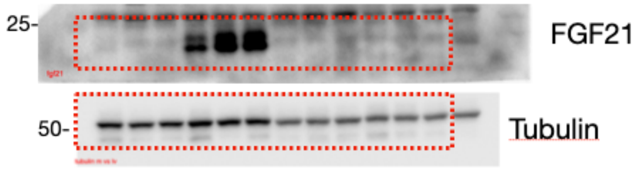

Figure 7H

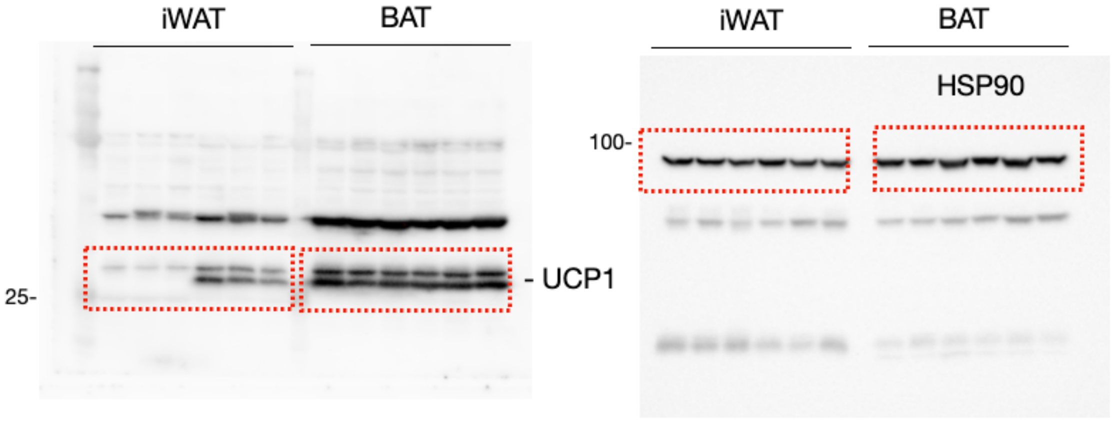

Figure 7E

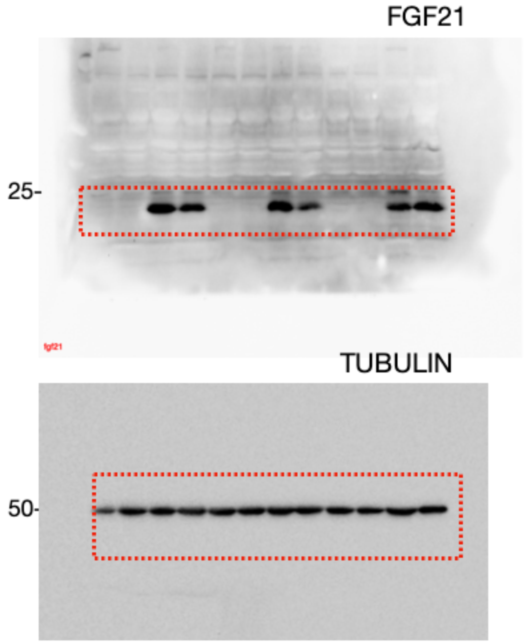

Figure 8G

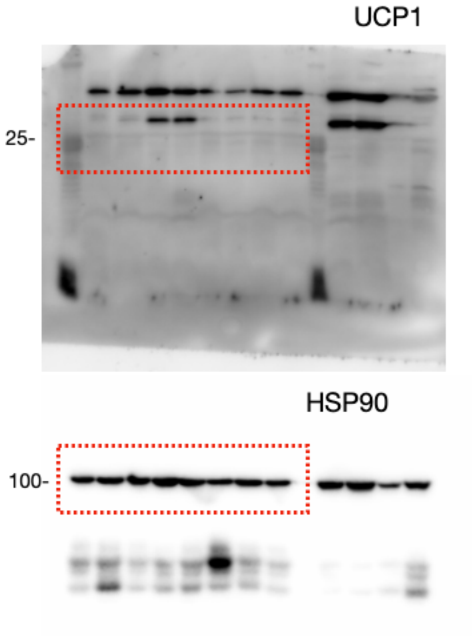

Figure 8A

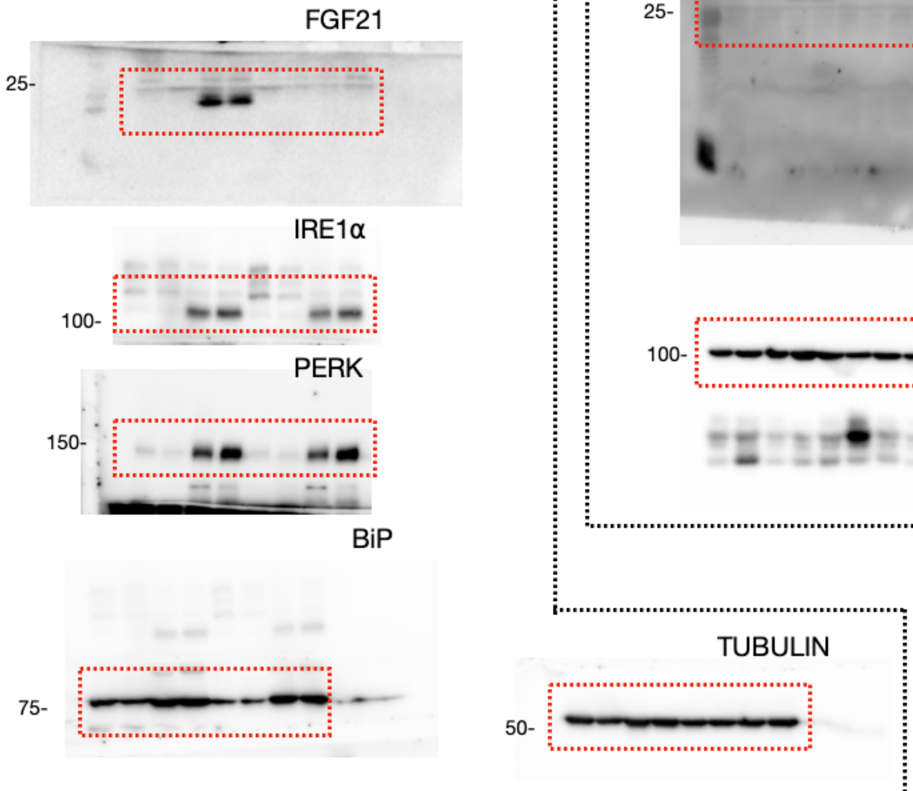

Supplement: Supplemental data [file jciinsight-8-170387-s009.pdf]
